# Multi‐Subunit Engineering of Photorespiratory Glycine Decarboxylase Refines Photosynthesis, Growth and Metabolic Robustness of *Arabidopsis*

**DOI:** 10.1111/pce.70836

**Published:** 2026-08-21

**Authors:** Hu Sun, Yannic C. R. Wagner, Wieke‐Marie Rosenfeldt, Rhena Schumann, Stéphanie Arrivault, Guillaume Decros, Rhiannon Page, Elizabete Carmo Silva, Tracy Lawson, Wei Huang, Alisdair R. Fernie, Hermann Bauwe, Martin Hagemann, Stefan Timm

**Affiliations:** ^1^ University of Rostock, Plant Physiology Department Albert‐Einstein‐Straße 3 Rostock Germany; ^2^ Biological Station Zingst, University of Rostock Zingst Germany; ^3^ Max Planck Institute of Molecular Plant Physiology, Am Mühlenberg 1 Golm Germany; ^4^ Lancaster Environment Centre, Lancaster University LA1 Lancaster UK; ^5^ University of Essex, Wivenhoe Park Colchester UK; ^6^ Yunnan Key Laboratory of Forest Ecosystem Stability and Global Change, Xishuangbanna Tropical Botanical Garden, Chinese Academy of Sciences Mengla Yunnan China

## Abstract

Photorespiration secures oxygenic photosynthesis from metabolic constraints arising from the oxygenation reaction of Rubisco in today's oxygen‐rich atmosphere. Beyond this protective function, it is vital for primary metabolism, cellular redox balance, and stomatal regulation. Importantly, photorespiration has emerged as a key target for improving CO_2_ assimilation and biomass production. Among proposed strategies, enhancing photorespiratory efficiency via upregulation of core enzymes has highlighted glycine decarboxylase (GDC) as a promising target, however, it remains unclear whether simultaneous upregulation of multiple GDC components would yield additive or synergistic improvements in plant environmental acclimation. Here, we systematically compared single and double GDC overexpression lines under varying photoperiods, elevated oxygen and high light. GDC upregulation consistently promoted growth across photoperiods and enhanced photosynthetic performance under high light and oxygen, indicating a severe benefit of increased photorespiratory metabolite conversion. Notably, double overexpression lines exhibited enhanced metabolic flexibility beyond that observed in single lines, supporting synergistic effects on carbon assimilation and allocation. Short‐term high‐light exposure further revealed increased Rubisco activity and efficient partitioning of carbon into sucrose and starch. Collectively, our results demonstrate that coordinated enhancement of GDC activity strengthens photorespiratory efficiency and can generate synergistic improvements in photosynthetic robustness, metabolic robustness, and environmental acclimation under stressful conditions. These findings position multicomponent GDC engineering as a promising strategy for developing crops with improved environmental acclimation capacity.

## Summary statement

Coordinated overexpression of glycine decarboxylase subunits in Arabidopsis enhances photorespiratory flux, improving photosynthesis, growth and stress resilience. Multicomponent engineering yields synergistic gains in carbon assimilation and metabolic flexibility beyond single‐subunit upregulation.

## 1 Introduction

Photorespiration is indispensable for oxygenic photosynthesis, safeguarding efficient CO_2_ assimilation and carbon utilization by preventing negative metabolic feedback originating from Rubisco's oxygenation reaction and the accumulation of its major by‐product, 2‐phosphoglycolate (2PG) (reviewed in Fernie and Bauwe 2020). Despite this essential protective role, intrinsic inefficiencies within the photorespiratory pathway cause a substantial yield penalty, particularly in C_3_ crops exhibiting high photorespiration (Walker et al. 2016; Eisenhut et al. 2019; Busch 2020). During 2PG detoxification, mitochondrial glycine decarboxylase (GDC) releases CO_2_ via the conversion of glycine to serine, resulting in carbon losses that can reduce net photosynthetic gain by ~25%–30% under standard conditions and up to ~50% under environments that promote photorespiration (Walker et al. 2016). In addition, photorespiration consumes significant amounts of ATP and NADPH, particularly during re‐assimilation of released ammonia via the glutamine synthetase/glutamate synthase (GS/GOGAT) cycle (Foyer et al. 2009; Walker et al. 2024). As climate change is expected to intensify photorespiratory pressure through increased temperatures, drought frequency, and light fluctuations, improving our understanding and optimization of photorespiration is critical for enhancing crop resilience (Walker et al. 2016; Cavanagh et al. 2022; Long et al. 2025).

Given the considerable energetic and carbon costs associated with photorespiration, significant effort has focused on engineering strategies to reduce photorespiratory inefficiencies (South et al. 2018; Chen et al. 2026; Meacham‐Hensold et al. 2026; Shen et al. 2019). One major approach involves the introduction of synthetic bypasses that minimize mitochondrial CO_2_ release and associated energy losses. Successful examples include chloroplast‐localized glycolate oxidation pathways, often combined with partial restriction of carbon influx into native photorespiration, thereby aiming to increase CO_2_ availability around Rubisco (Pick et al. 2013; Xin et al. 2015; South et al. 2019). Building on pioneering work (Kebeish et al. 2007), these strategies have been extended to crop species such as tobacco and potato and validated under field conditions, resulting in enhanced photosynthesis, biomass accumulation, and improved tolerance to heat stress (South et al. 2019; Cavanagh et al. 2022; Meacham‐Hensold et al. 2024, 2026). An alternative strategy focuses on optimizing endogenous photorespiratory efficiency rather than bypassing it. This approach is based on the premise that increasing the turnover of photorespiratory intermediates alleviates metabolic feedback on the Calvin‐Benson‐Bassham cycle (CBBC) and downstream carbon metabolism (Timm et al. 2012; Flügel et al. 2017; Timm and Hagemann 2020), while preserving the pathway's central role in primary metabolism, nitrogen assimilation, redox balance, and energy homeostasis (Foyer et al. 2009; Eisenhut et al. 2017; Smith et al. 2024; Walker et al. 2024). In this context, GDC has emerged as a key regulatory node. Overexpression of genes encoding the GDC H‐ and l‐proteins enhanced photosynthesis and growth in Arabidopsis and tobacco (Timm et al. 2012, 2015; Simkin et al. 2017; López‐Calcagno et al. 2019), and additive effects have been reported when GDC upregulation is combined with enhanced CBBC activity (Timm et al. 2016; Simkin et al. 2017). Similarly, increasing 2PG turnover via 2PG phosphatase 1 (PGLP1) improves photosynthetic performance and metabolic output under elevated photorespiratory pressure (Flügel et al. 2017; Timm et al. 2019). While overexpression of additional enzymes, including glycolate oxidase (GOX), the plastidal glycolate:glycerate transporter 1 (PLGG1), and serine hydroxymethyltransferase (SHMT1), have also been targeted in different species, their effects were largely condition‐specific and generally do not reach the magnitude of photosynthetic and growth enhancement achieved through GDC manipulation (Moreno et al. 2005; Cui et al. 2016, 2025; Liu et al. 2019; Timm et al. 2026). In other cases, photorespiratory enzyme overexpression has shown neutral or even detrimental effects on plant growth and metabolism (Igarashi et al. 2006; Modde et al. 2017; Timm et al. 2018). Together, these findings identify GDC as a major determinant of photorespiratory efficiency, that is, the internal metabolite turnover. However, while individual components of the GDC complex have been successfully targeted, it remains, as yet, unclear if simultaneous upregulation of multiple GDC components would result in merely additive or truly synergistic improvements of photorespiration and plant performance under environmental challenges.

Given the central role of GDC in coordinating photorespiration and associated metabolic pathways we tested the hypothesis if combined overexpression of the GDC H‐ and l‐proteins leads to synergistic enhancements in photosynthetic efficiency, growth, and metabolic flexibility compared to single overexpression lines. To address this, we systematically compared single and double GDC overexpression lines under standard conditions as well as across a range of environmental regimes, including altered photoperiods, elevated oxygen and high light. Through integrated physiological and metabolic analyses, we assessed whether coordinated increases in GDC activity improves photorespiratory efficiency and confers improved environmental acclimation and metabolic robustness under conditions relevant to future climate scenarios.

## 2 Materials and Methods

### 2.1 Plant Material, Standard Growth Conditions and Biomass Quantification

Columbia.0 (Col.0) served as the *Arabidopsis thaliana* (*Arabidopsis*) wild‐type reference throughout this study. Previously described single GDC overexpression lines, FpH18 (stable GDC‐H overexpression; Timm et al. 2012) and PsL2 (stable GDC‐L overexpression; Timm et al. 2015), were crossed to generate the double overexpression line H18L2. Seeds were surface‐sterilized with chloric acid, sown on a soil (Type Mini Tray; Einheitserdewerk, Uetersen, Germany) and vermiculite mixture (4:1), and stratified at 4°C for at least 2 days to break dormancy. Plants were grown in controlled‐environment chambers (Percival) under standard conditions: a 12 h light/12 h dark photoperiod, 22/20°C day/night temperatures, ~120–150 µmol m^−2^ s^−1^ light intensity, 400 ppm CO_2_, 21% O_2_, and ~70% relative humidity. For specific experiments, the photoperiod was adjusted to 10 h light/14 h dark or 14 h light/10 h dark. Long‐day cultivation was additionally performed in a greenhouse during the winter season. Plants were fertilized regularly with 0.2% Wuxal (Aglukon, Düsseldorf, Germany). All physiological experiments were conducted on randomly grown plants at stage 5.1 (Boyes et al. 2001). Quantitative growth parameters were determined from 8 to 12 biological replicates per genotype, including: (i) rosette diameter (maximum distance across the fully expanded rosette), (ii) total leaf number (counting only fully expanded rosette leaves), (iii) fresh weight (FW), (iv) dry weight (DW) of entire rosettes (dried for ~24 h at 100°C), and (v) the FW/DW ratio.

### 2.2 Plant High Light Treatment

Short‐term high‐light treatments were performed in controlled‐environment chambers (Conviron). Plants were initially grown under standard conditions under normal light (NL; ~120 µmol m^−2^ s^−1^) and then transferred at the end of the night (EoN; prior to the onset of illumination) to a second chamber with increased light intensity (500 µmol m^−2^ s^−1^; high light, HL), while all other conditions were maintained constant. Whole plants were harvested at EoN and after 4, 8 and 12 h under both, NL and HL conditions. Samples were immediately flash‐frozen in liquid nitrogen and stored at −80°C until further analysis. Prior to the quantification of selected primary metabolites (ammonia, starch, sucrose, 3PGA, amino acids, and organic acids), plant material was freeze‐dried (lyophilized) to facilitate aliquoting for subsequent analyses.

### 2.3 PCR Verification of the Transgenic Lines and Immunological Studies

The presence of the overexpression constructs was verified via PCR (1 min at 94°C; 1 min at 58°C and 1–2 min at 72°C; 35 cycles), using leaf DNA as template. For this purpose, we used oligonucleotides that specifically bind the ST‐LSI promoter (P281; 5′‐AACAACCCATAGAGGAACCAATCA‐3′) in combination with either, the *Fp*GDCH (P966; 5′‐CTGCAGCTACGTGAGCAGAATCTTCTTC‐3′) or the *Ps*GDC‐L (P294; 5′‐GAATTCTCAAATGTGAATGGGCTTGTC‐3′) sequences. The *S16* gene was amplified (1 min at 94°C, 1 min at 58°C and 30 s at 72°C; 35 cycles) using oligonucleotides *S16*‐forward (P444; 5′‐GGCGACACAACCAGCTACTGA‐3′) and *S16*‐reverse (P445; 5′‐CGGTAACTCTTCTGGTAACGA‐3′) as control (see Figure S1).

Overexpression of selected photorespiratory proteins was analyzed by immunoblotting. Briefly, whole‐leaf proteins were separated by SDS‐PAGE and gel blotting experiments performed according to standard protocols. Changes in the protein abundances were detected using specific antibodies for the glycine decarboxylase (GDC) proteins GDC‐H, GDC‐L, GDC‐P, GDC‐T, serinehydroxymethyltransferase 1 (SHMT1), PGLP1 (Timm et al. 2013) and SBPase (Driever et al. 2017). Were stated, ImageJ was used to determine the band intensities as a measure of altered protein abundances from at least three independent immunoblots.

### 2.4 Guard Cell (GC) Properties and GC Starch Content

Diagnostic parameters associated with GC morphology were determined using epidermal peels prepared from plants grown under standard conditions. Peels were harvested during the light period (~6 h of illumination) following the application of transparent nail polish to the abaxial leaf surface. After drying (10 min), the epidermis was peeled off, transferred to water on microscope slides, and covered with a coverslip. GC parameters (area, length, width, density, and index) were determined from at least four biological replicates using an Olympus U‐LH100HG microscope (Olympus Corporation, Japan) in combination with the manufacturer's image analysis software. Starch content in GC was assessed in epidermal peels harvested at midday (~6 h of illumination) following propidium iodide staining, as described previously (Flütsch et al. 2018; Sun et al. 2026). At least four biological replicates per genotype were analyzed, with 10 randomly selected GC per stained peel.

### 2.5 Chlorophyll Fluorescence and Gas Exchange Measurements

Chlorophyll fluorescence and gas exchange measurements were performed on plants grown under environmentally controlled conditions (Percival growth chamber; 12 h light/12 h dark cycle; ~120 µmol m^−2 ^ s^−1^ irradiance) until developmental stage 5.1 (Boyes et al. 2001). Photosystem II (PSII)‐ and Photosystem I (PSI)‐related parameters were determined using a Dual‐PAM‐100 (Heinz Walz, Effeltrich, Germany). Leaf gas exchange was measured using LI‐6400XT and LI‐6800 Portable Photosynthesis Systems (LI‐COR Biosciences, Lincoln, NE, USA). All measurements were conducted on side‐by‐side plants grown of the same experimental series and as previously described (Sun et al. 2025).

### 2.6 Determination of (Non‐)Phosphorylated Primary Metabolites

For the absolute quantification of selected diagnostic metabolites involved in primary metabolism, gas chromatography (GC) and liquid chromatography coupled to tandem mass spectrometry (LC‐MS/MS) were employed. Fully expanded rosettes were harvested under growth light conditions at the end of the photoperiod (after 11 h of illumination). To minimize experimental variation, samples were collected within a 10 min interval, immediately quenched in liquid nitrogen, and stored at −80°C. Prior to further processing, samples were lyophilized, and approximately 2–3 mg of dry leaf material per sample was aliquoted. Metabolite extraction and LC‐MS/MS analyses were performed using LC‐MS grade reagents following previously described protocols (Arrivault et al. 2009; Reinholdt et al. 2019). Measurements were conducted using a UHPLC‐MS/MS‐8050 system (Shimadzu, Japan) equipped with the LC‐MS/MS method package for primary metabolites (version 2, Shimadzu; Reinholdt et al. 2019), or alternatively, using a Dionex HPLC system coupled to a Finnigan TSQ Quantum Discovery triple quadrupole mass spectrometer (Thermo Scientific) equipped with an electrospray ionization (ESI) interface (Arrivault et al. 2009, 2015). Selected soluble sugars and starch were quantified from the same extracts using a 6890 N GC System (Agilent Technologies, Waldbronn, Baden‐Württemberg, Germany) in combination with spectrophotometric assays, essentially as described previously (Sun et al. 2025). Absolute metabolite concentrations were determined based on calibration curves generated from authentic standards analyzed in parallel.

### 2.7 Spectrophotometric Determination of Ammonia

Ammonia was determined in water extracts prepared from approximately ~5 mg plant dry mass (dissolved in 400 µL H_2_O, 10 min at 80°C) after reacting with phenole and nitroprusside in the presence of Cl_2_ (from hypochlorite) to indophenolblue (Koroleff 1969) partly modified for a flow analyzer (Grasshoff and Johannsen (1972). Extracts were diluted by 100 to 200 times to reach the linear measuring range and to provide the required sample volume. The limit of quantification was 0.4 µmol L^−1^. The combined standard uncertainty of standards and replicate samples was 10.9%.

### 2.8 Rubisco Activity and Protein Measurements

Leaf proteins were extracted from samples consisting of 4 discs (1.38 cm^2^) taken from illuminated leaves (12 h) of plants grown under normal light (~120–150 µmol m^−2^ s^−1^) or high light (500 µmol m^−2^ s^−1^). Leaf samples were rapidly frozen in liquid nitrogen and stored at −80°C until extraction in 250 µL extraction buffer per sample exactly as described before (Ashton et al. 2024). Upon protein extraction, Rubisco activities in each sample were immediately measured using radiometric ^14^CO_2_ assays as previously described (Ashton et al. 2024). Briefly, an aliquot of supernatant from the leaf extract was added to an assay mix with ribulose‐1,5‐bisphosphate (RuBP) to give an estimate of Rubisco activity in the leaf at the time of sampling (initial activity, V_i_). A further aliquot was incubated in assay mix minus RuBP for 3 min to allow full Rubisco carbamylation prior to the addition of RuBP, quantifying the activity of all available Rubisco active sites (total activity, *V*_t_). Rubisco activation state was calculated as the ratio of *V*_i_ to *V*_t_. The remaining supernatant aliquot was used to quantify total soluble protein content using the Bradford method (Bradford 1976).

### 2.9 Software and Statistical Analysis

Data processing and graph generation were performed using Microsoft Excel (Microsoft Corporation, 2018) and SigmaPlot (Systat Software Inc., 2014). Image compilation was carried out using CorelDRAW Graphics Suite (Corel Corporation). Statistical analyses were conducted using IBM SPSS Statistics. Differences between groups were assessed using Student's *t*‐test or two‐way analysis of variance (ANOVA), followed by Tukey's honestly significant difference (HSD) and least significant difference (LSD) post hoc tests, as appropriate. Differences were considered statistically significant at *p* < 0.05.

## 3 Results

### 3.1 Generation and Verification of the GDC‐H and GDC‐L Double Overexpression Line

Simultaneous overexpression of the GDC H‐ and l‐proteins was achieved by crossing *Arabidopsis* lines with stable increases in GDC‐H (FpH18; Timm et al. 2012) and GDC‐L (PsL2; Timm et al. 2015), generating the double overexpression line H18L2. Our analysis was restricted to the cross between these two transgenic lines, as previous studies showed that they exhibited the strongest and most stable physiological responses. Both, lower and higher levels of GDC H‐ and l‐protein expression, respectively, diminish the beneficial effects of GDC overexpression and can even negatively affect plant performance (Timm et al. 2012, 2015; López‐Calcagno et al. 2019). Following genotypic confirmation, increased accumulation of GDC‐H and GDC‐L proteins in leaves of six individual crosses was verified by immunoblot analysis using specific antibodies (Figure S1). Next, protein accumulation was quantified in more detail by densitometry. Relative to wild type, GDC‐H (in FpH18 and H18L2) and GDC‐L (in PsL2 and H18L2) protein abundances increased approximately threefold in the mesophyll, whilst the control proteins PGLP1 and SBPase remained unchanged across all genotypes (Figure 1A). Combined overexpression did not affect the abundance of the other GDC subunits (P and T), and remained comparable between the double mutant H18L2 and the single lines (Figure 1A).

**Figure 1:**
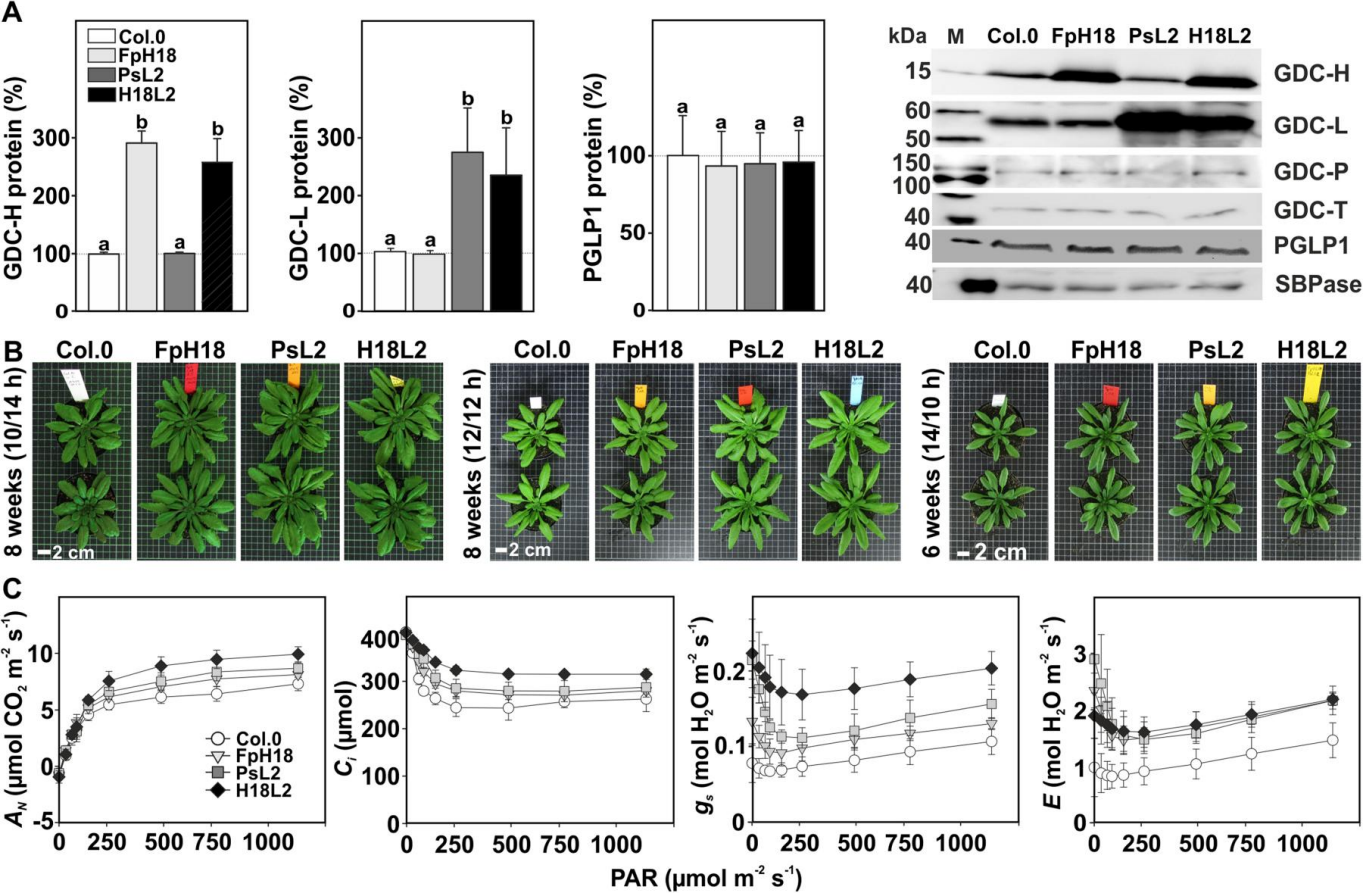
Protein abundance, phenotype, and gas exchange in transgenic lines compared to wild type. (A) GDC‐H, GDC‐L, and PGLP1 protein abundance in leaves of plants at growth stage 5.1 (Boyes et al. 2001). Proteins were extracted as pools from at least 3 biological individuals, detected by immunoblotting and quantified by densitometry. Values are expressed as % band intensity relative to wild type (Col.0 was set to 100%; values are means ± SD, *n* = 3; independent technical replicates). Representative blots of GDC‐H (15.23 kDa), GDC‐L (54.00 kDa), GDC‐P (112.92 kDa), and GDC‐T (46.35 kDa) are displayed in addition and in comparison, with the two control proteins PGLP1 (39.76 kDa) and SBPase (42.41 kDa). M, Protein marker, kDa, kilodalton. (B) Representative phenotypes of *Arabidopsis* WT, FpH18, PsL2, and H18L2 grown under short‐day (10/14 h, left), mid‐day (12/12 h, middle), and long‐day (14/10 h, right, greenhouse) conditions (for quantitative biomass data see Table S1). (C) Gas exchange parameters under standard growth conditions (12/12 h photoperiod): net CO_2_ assimilation rate (*A*_*N*_), intercellular CO_2_ concentration (*C*_*i*_), stomatal conductance (*g*_*s*_), and transpiration rate (*E*). Data represent mean ± SD (*n* = 6; independent biological replicates). Different letters indicate significant differences (ANOVA). All numerical values and the statistical evaluation are provided as Table S3.

### 3.2 GDC Overexpression Consistently Increases Growth Across Photoperiods

To assess growth performance, phenotypic and biomass analyses were conducted for the GDC double overexpression line H18L2 under different photoperiods. Consistent with previous reports, the single overexpression lines FpH18 and PsL2 displayed visibly enhanced growth relative to wild type (Figure 1B). In contrast, growth of the double mutant H18L2 was also stimulated, but not further increased compared to the single overexpression lines under any tested photoperiod (Figure 1B). However, increased growth became apparent from week three of the growth cycle and remained stable until the end of the growth period. This visual observation was supported by quantitative measurements. Rosette diameter, total leaf number, fresh weight, dry weight, and FW/DW ratios did not differ significantly between single and double overexpression lines. However, again, all of these parameters were significantly elevated in all GDC overexpressors compared to wild type and in all photoperiods (Table S1).

### 3.3 Surplus of Photosynthetic Electron Transport and CO_2_ Assimilation Enhancement in H18L2 Is High Light‐Dependent

To determine whether GDC overexpression affects photosynthetic light reactions, chlorophyll fluorescence and P700 measurements were performed across increasing light intensities. The maximum quantum efficiency of PSII (*F*_*v*_/*F*_*m*_) and the maximal P700 oxidation signal (*P*_*m*_), reflecting PSI capacity, were largely unchanged among genotypes under standard growth conditions, although a modest but significant increase in *P*_*m*_ was observed in H18L2. Analysis of the effective quantum yields of PSII [Y(II)] and PSI [Y(I)] revealed no differences at low irradiance. However, under high light, both parameters were significantly increased in H18L2 compared to wild type and the single lines. Consistently, relative electron transport rates showed a pronounced enhancement in H18L2, with rETR(II) elevated across a broad high‐light range and rETR(I) increasing significantly at irradiances above 750 µmol m^−2^ s^−1^. In contrast, single overexpression lines remained comparable to wild type (Figure S2 and Table S1). Parameters associated with energy dissipation and PSI limitation, including non‐photochemical quenching (NPQ), cyclic electron flow (CET), and PSI acceptor‐ and donor‐side limitations [Y(NA) and Y(ND)], were largely unaffected across genotypes. Only a slight but significant reduction in Y(ND) was seen in H18L2 at irradiances ≥ 500 µmol m^−2^ s^−1^ (Figure S2 and Table S2). Together, these results indicate that enhanced photosynthetic electron transport is specifically induced in H18L2 under high light conditions.

To assess whether these changes translate into improved carbon assimilation, gas exchange measurements were conducted under increasing light intensities. Net CO_2_ assimilation (*A*_*N*_) was significantly higher than wild type in the single overexpression lines at irradiances ≥ 237 µmol m^−2^ s^−1^, whereas H18L2 exhibited increased *A*_*N*_ already at ≥ 141 µmol m^−2^ s^−1^ (Figure 1C), indicating an earlier and stronger photosynthetic response. Intercellular CO_2_ concentration (*Cᵢ*), stomatal conductance (*g*_*s*_), and transpiration (*E*) were elevated in all overexpression lines, with the strongest increases consistently observed in H18L2 (Figure 1C). In contrast, intrinsic water‐use efficiency was unchanged in the single lines but significantly reduced in H18L2 (Table S3). Taken together, these data demonstrate that only the combined overexpression of GDC‐H and GDC‐L leads to a pronounced, high light‐dependent enhancement of *photosynthetic* electron transport that is coupled to increased CO_2_ assimilation. The comparatively smaller effects observed in the single overexpression lines suggest that the GDC components act synergistically to enhance photosynthetic performance under high irradiance.

### 3.4 GDC Overexpression Enhances Photosynthesis and Reduces O_2_ Sensitivity, With Strongest Effects in H18L2

To determine whether enhanced CO_2_ assimilation in GDC overexpression lines is associated with altered photorespiratory flux, O_2_‐dependent CO_2_ response curves were measured at low (3%), ambient (21%), and elevated (40%) O_2_ concentrations to modulate photorespiratory activity. Consistent with previous studies (Timm et al. 2012, 2015), both single overexpression lines exhibited improved photosynthetic performance under photorespiratory conditions, including increased *A*_*N*_, *g*_*s*_, *C*_*i*_, and *E*, along with a reduced CO_2_ compensation point (*Γ*) compared to wild type (Figure 2A–D and Table S4). These differences were negligible at low O_2_ (3%), where photorespiration is largely suppressed, but became increasingly pronounced at elevated O_2_ (40%), which promotes Rubisco oxygenation. Notably, the double overexpression line H18L2 consistently showed further increases in these parameters relative to both single lines, indicating enhanced photosynthetic performance under conditions of high photorespiratory pressure. Further, O_2_ inhibition of *A*_*N*_ was reduced in all overexpression lines, with the strongest effect observed in H18L2. Furthermore, the slope of the *Γ*‐O_2_ relationship (*γ*), reflecting the stoichiometry of photorespiratory CO_2_ release, was decreased compared to wild type (Table S5), supporting that improved photorespiratory efficiency did not cause higher photorespiratory CO_2_ release. Together, these results demonstrate that GDC overexpression enhances photosynthesis and reduces the O_2_ sensitivity of CO_2_ assimilation, with the most pronounced effects observed in the double overexpression line, consistent with a synergistic enhancement of photorespiratory efficiency.

**Figure 2:**
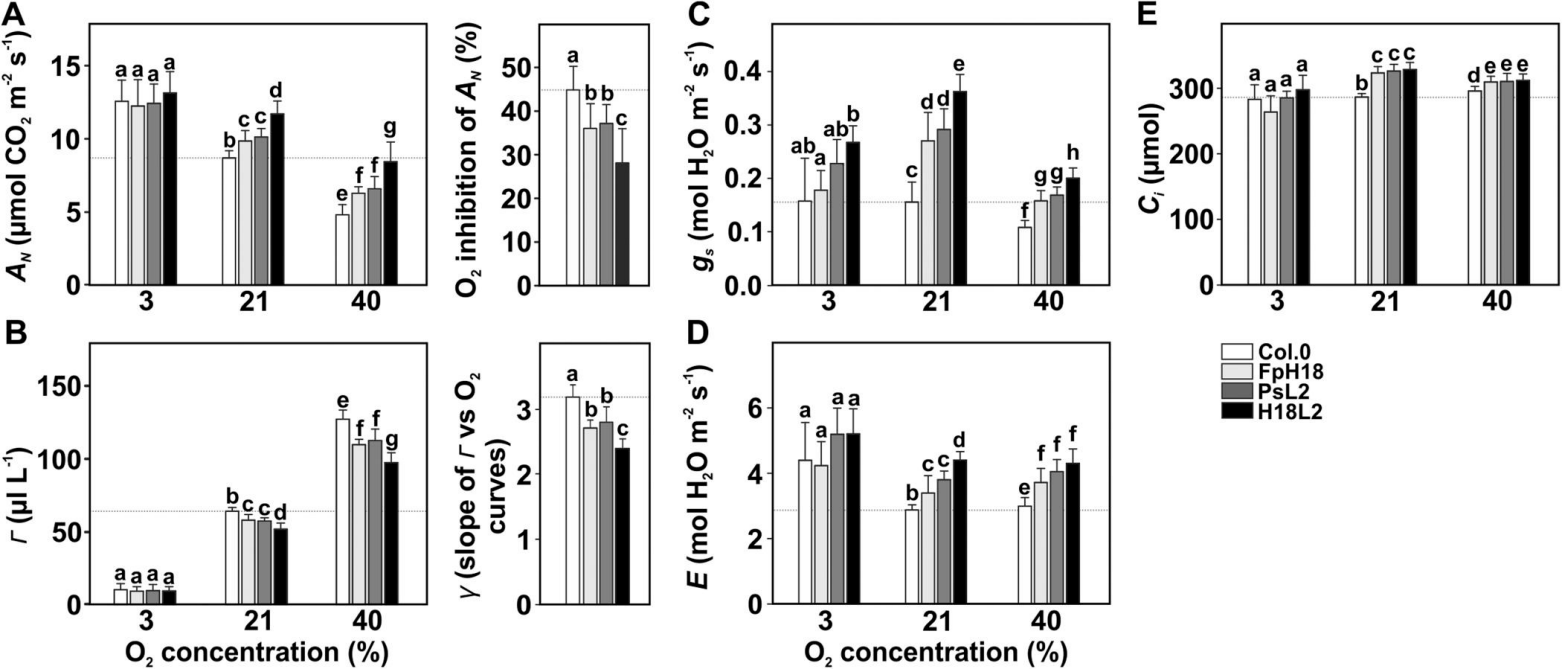
O_2_‐dependent steady‐state leaf gas exchange parameters of GDC overexpression lines and the wild type. Selected gas exchange parameters were determined at three different O_2_ concentrations (3%, 21%, and 40%) using plants grown under standard conditions to stage 5.1 (Boyes et al. 2001). Displayed are means ± SD (*n* = 6; independent biological replicates) of: (A) net CO_2_ assimilation rates (*A*_*N*_) and O_2_ inhibition of *A*_*N*_; (B) CO_2_ compensation points (*Γ*) and slopes of the *Γ*‐vs‐O_2_ curves (*γ*); (C) stomatal conductance (*g*_*s*_); (D) transpiration rates (*E*); and (E) intracellular CO_2_ concentrations (*C*_*i*_). Values that do not share the same letter are significantly different from each other based on ANOVA analysis. Full numerical data is given as Tables S4 and S5. ANOVA, analysis of variance; GDC, glycine decarboxylase.

### 3.5 GDC Overexpression Does Not Alter Stomatal Morphology but Enhances Starch Accumulation in GC and Leaves

The observed increases in *g*_*s*_ in GDC overexpression lines (Figures 1C and 2C) prompted us to investigate whether these changes were associated with alterations in stomatal morphology or GC metabolism. Consistent with previous findings on GC‐specific GDC manipulation (Sun et al. 2025), no significant differences were detected in stomatal density, size, area, or stomatal index among the analyzed genotypes (Table 1), indicating that GDC overexpression does not affect stomatal development. However, in well agreement with higher *g*_*s*_ in GDC‐manipulated lines, stomatal aperture was also increased. Further, all GDC overexpression lines exhibited significantly increased GC starch accumulation. Notably, this effect was not restricted to GC but was also observed at the whole‐leaf level under standard growth conditions (Table 1). These results indicate that enhanced *g*_*s*_ in GDC overexpression lines was not driven by structural changes in stomatal anatomy but was instead associated with altered carbon metabolism, including increased starch biosynthesis in both GC and leaf tissue.

**Table 1:** Stomatal parameters and starch in GDC overexpression lines and the wild type. Plants were grown under environmental controlled conditions to growth stage 5.1 (Boyes et al. 2001). Given stomata‐related parameters are means ± SD of at least 8 independent biological replicates, of which 15 stomata per leaf were evaluated (120 stomata in total). Guard cell starch (*n* = 4, independent biological replicates; 10 stomata per leaf, Σ = 40) and whole leaf starch contents (*n* = 6; independent biological replicates) were determined from the same plants at end of the day (11 h illumination). Values that do not share the same letter are significantly different from each other as determined by ANOVA analysis.

Parameter	Genotype			
	Col.0	FpH18	PsL2	H18L2
Stomatal density (µm^−2^)	98.4 ± 8.1^a^	99.7 ± 9.4^a^	98.4 ± 12.9^a^	103.3 ± 19.3^a^
Long axis (μm)	18.7 ± 1.1^a^	18.8 ± 0.9^a^	18.5 ± 1.1^a^	18.5 ± 0.9^a^
Short axis (μm)	14.0 ± 0.7^a^	13.7 ± 0.5^a^	14.0 ± 0.7^a^	14.6 ± 0.5^a^
Stomatal area (μm^−2^)	411.7 ± 40.0^a^	403.8 ± 28.4^a^	407.7 ± 38.8^a^	424.2 ± 34.4^a^
Stomatal index (mm^−2^ mm^−2^)	0.04 ± 0.007^a^	0.04 ± 0.003^a^	0.04 ± 0.005^a^	0.04 ± 0.010^a^
Stomatal aperture (µm)	7.5 ± 1.6^a^	12.4 ± 2.1^b^	13.1 ± 1.5^b^	14.1 ± 1.5^b^
Starch				
GC (area per stomata in μm^2^)	11.8 ± 2.1^a^	15.4 ± 0.4^b^	15.9 ± 0.8^bc^	17.5 ± 1.2^c^
Leaves (μmol g^−1^)	45.0 ± 8.7^a^	53.8 ± 10.7^ab^	53.0 ± 7.8^b^	55.0 ± 5.7^b^

Abbreviations: ANOVA, analysis of variance; GDC, glycine decarboxylase.

### 3.6 Metabolite Profiling Reveals Enhanced Photorespiratory Metabolite Turnover and Reprogramming of Carbon Allocation Toward Amino Acids and Soluble Sugars

To answer the question if modified photosynthetic performance and stomatal conductance are associated with metabolic changes, targeted metabolite profiling was performed on plants grown under standard conditions. Soluble sugars were quantified by gas chromatography, while amino acids, organic acids, and phosphorylated intermediates were analyzed by liquid chromatography coupled to tandem mass spectrometry (LC‐MS/MS). GDC overexpression led to pronounced shifts in primary metabolism (Figure 3A–C). All overexpression lines exhibited significantly increased levels of total amino acids (19 proteinogenic amino acids; Table S6) and soluble sugars (sucrose, glucose and fructose) compared to wild type, whereas total organic acid content remained largely unchanged. In parallel, ADP‐glucose (ADPG) levels were significantly reduced in the transgenic lines, while UDP‐glucose (UDPG) remained unaffected (Figure 3D,E), indicating altered carbon partitioning within central metabolism. Consistent with enhanced photorespiratory efficiency, key intermediates of the pathway, including 2PG, glycine, and glycerate, were decreased in all GDC overexpression lines (Figure 3F–H). Although serine levels remained unchanged, the Gly/Ser ratio, a proxy for efficient photorespiratory metabolite conversion, was significantly reduced, suggesting increased pathway efficiency (Figure 3I).

**Figure 3:**
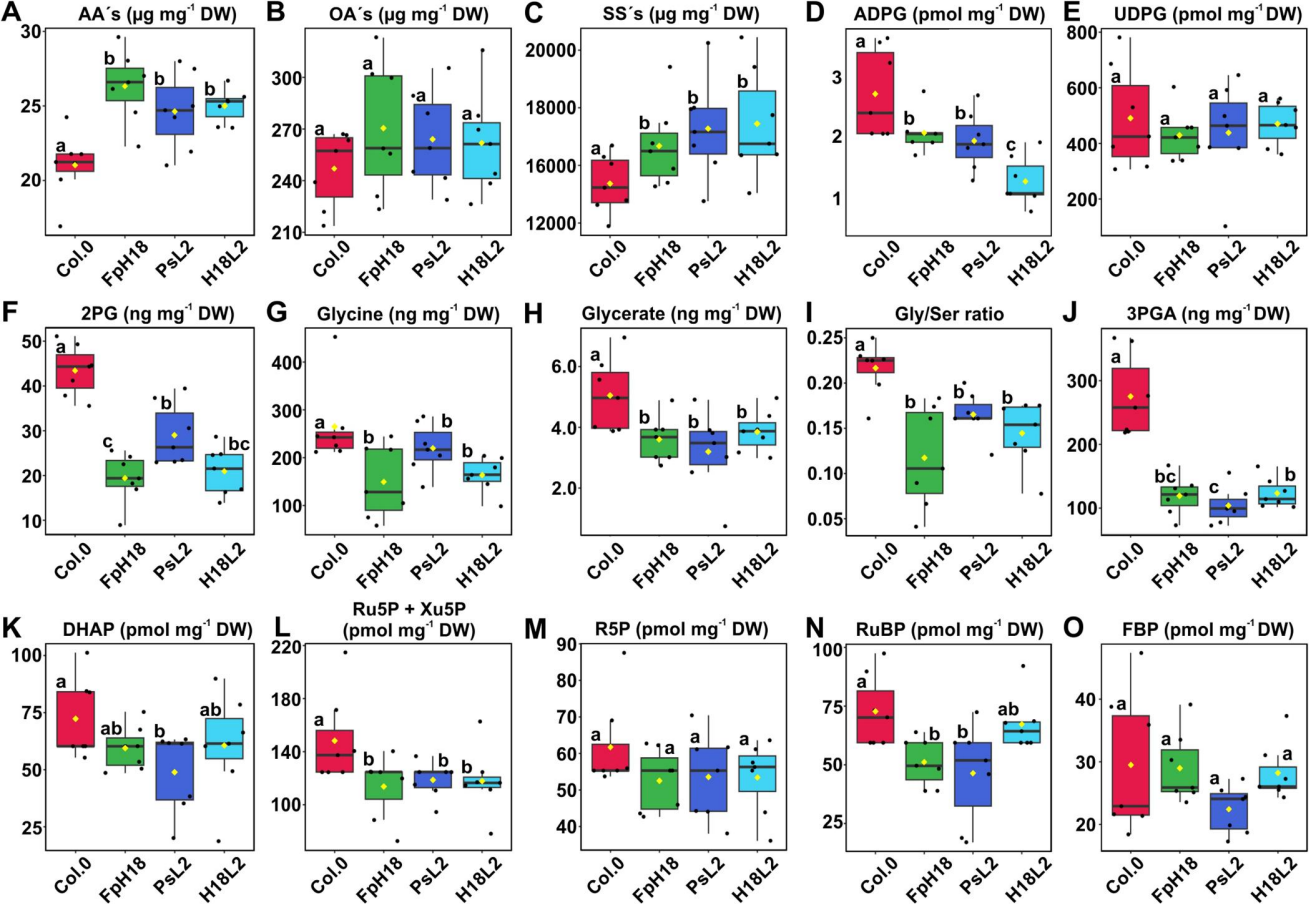
Absolute metabolite abundances in GDC overexpressors and the wild type. Plants were grown under environmental controlled conditions (12/12 h day/night cycle; 400 ppm CO_2_; 6 weeks) to growth stage 5.1 (Boyes et al. 2001) and leaf‐material harvested at the end of the day (11 h illumination). Given metabolites are means ± SD (*n* = 5; independent biological replicates). Values that do not share the same letter are significantly different from the wild type as determined by ANOVA (*p* < 0.05). For full numerical data and further information see Table S6. AA's, total amino acids; OA's, total organic acids; SS's, soluble sugars. [Color figure can be viewed at wileyonlinelibrary.com]

Within the CBBC, only a subset of intermediates exhibited consistent changes. Notably, 3‐phosphoglycerate (3PGA), dihydroxyacetone phosphate (DHAP), ribulose‐5‐phosphate + xylulose‐5‐phosphate (Ru5P + Xu5P), and ribulose‐1,5‐bisphosphate (RuBP) showed lower absolute abundances resembling those observed for photorespiratory intermediates, while ribose‐5‐phosphate (R5P) displayed only a similar, non‐significant, trend (Figure 3J–N). In contrast, most other CBBC intermediates and associated metabolites, including fructose‐6‐phosphate (F6P), fructose‐1,6‐bisphosphate (FBP), glucose‐1‐phosphate (G1P), glucose‐6‐phosphate (G6P), sedoheptulose‐7‐phosphate (S7P), sedoheptulose‐1,7‐bisphosphate (SBP), and shikimate, remained unaltered (Figure 3O and Table S6). In summary, these data suggest that GDC overexpression improves photorespiratory metabolite conversion and selectively reconfigures central carbon metabolism, promoting carbon allocation toward amino acid and soluble sugar biosynthesis while maintaining overall metabolic robustness.

### 3.7 Enhanced Photorespiratory Metabolism Reshapes Metabolic Robustness and Carbon Allocation During High Light Exposure

To further investigate how optimized photorespiration influences carbon metabolism under conditions that strongly stimulate photorespiration, that is, 2PG biosynthesis, we performed a short‐term high light acclimation experiment. Based on the observed increases in photosynthetic performance and electron transport under elevated light (Figures 1 and S2) and enhanced CO_2_ assimilation under photorespiratory conditions (Figure 2), plants were shifted from normal light (~120 µmol m^−2^ s^−1^) to high light (500 µmol m^−2 ^s^−1^). Whole rosettes were harvested at the end of night (EoN) and after 4, 8 and 12 h of normal and high light to quantify key metabolites of primary metabolism. Ammonia levels remained stable across genotypes and conditions (Figure 4A), indicating that enhanced photorespiratory efficiency did not lead to detectable imbalances in nitrogen re‐assimilation, that is, the photorespiratory nitrogen cycle. In contrast, significant changes were observed in carbon metabolism. Sucrose (Figure 4B) and starch (Figure 4C) increased progressively during illumination, reaching significantly higher levels in GDC overexpression lines. Under standard light, this increase was most evident in PsL2 and H18L2 after 12 h, whereas under high light all overexpression lines exhibited elevated carbohydrate levels (Figure 4B,C), consistent with enhanced carbon assimilation and allocation.

**Figure 4:**
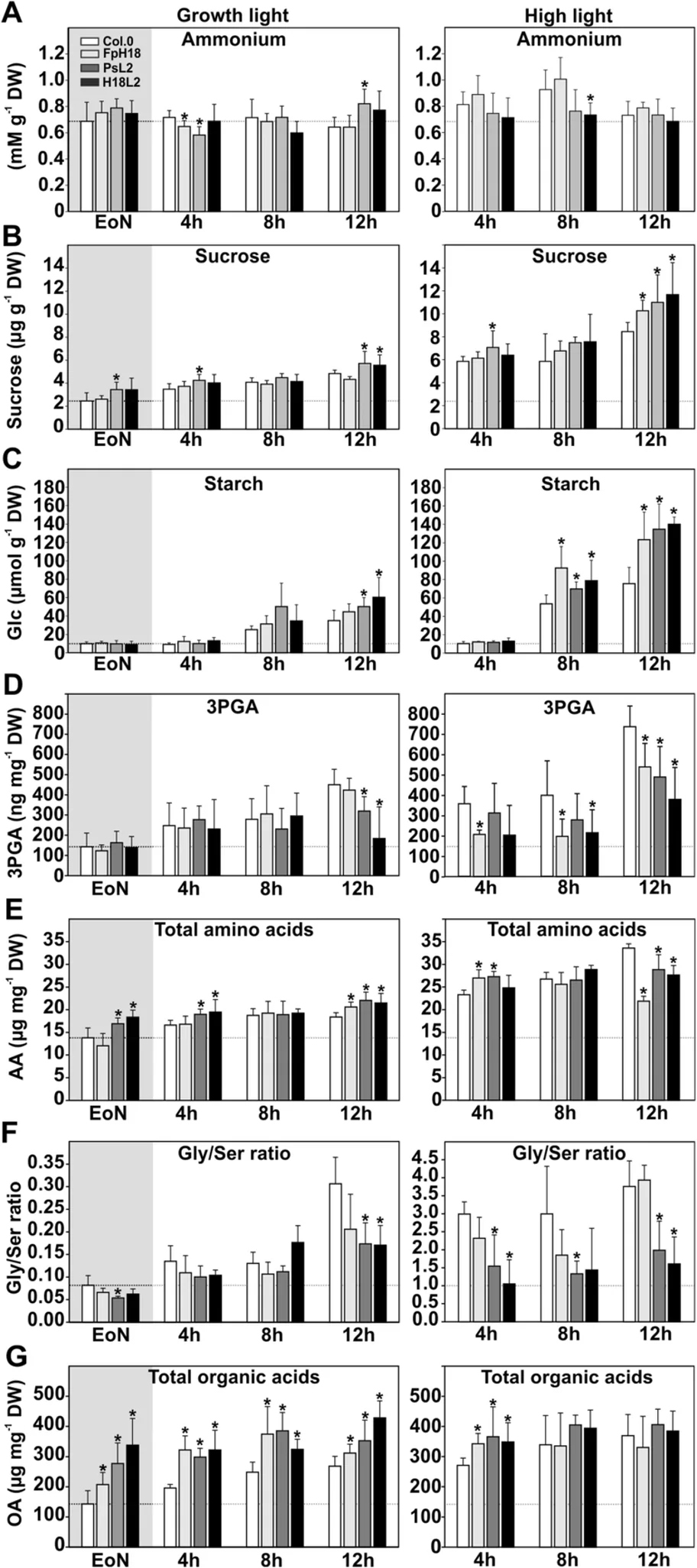
Absolute metabolite abundances in GDC overexpressors and the wild type in normal and high light. Plants were grown under environmental controlled conditions (~120 µmol m^−2^ s^−1^ normal light [NL]; 12/12 h day/night cycle; 400 ppm CO_2_; 6 weeks) to growth stage 5.1 (Boyes et al. 2001) and shifted to high light (500 µmol m^−2^ s^−1^ high light [HL], with otherwise equal conditions). Leaf‐material was harvested at the end of the night (EoN; 11 h darkness) and after 4, 8 and 12 h of NL and HL illumination. Given metabolites are means ± SD (*n* = 5, independent biological replicates). Values marked with asterisks are significantly different from the wild type as determined by Students *t*‐test (*p* < 0.05). For full numerical data and further information see Table S7.

In parallel, 3PGA levels remained relatively stable under standard light but declined in GDC overexpression lines after prolonged illumination, particularly in PsL2 and H18L2. This decrease was more pronounced under high light, where 3PGA levels were consistently lower across most time points (Figure 4D), suggesting increased metabolic turnover downstream of the CBBC. Total amino acid levels were elevated in GDC overexpression lines early in the photoperiod under both light regimes, particularly in PsL2 and H18L2. However, under prolonged high light exposure, amino acid levels declined and were significantly lower than in wild type after 12 h (Figure 4E), indicating a temporal shift in carbon allocation away from nitrogen‐containing compounds toward carbohydrate storage. Consistently, Gly/Ser ratios, a proxy for photorespiratory capacity, were generally reduced in GDC overexpression lines across conditions, with strongest effects in PsL2 and H18L2 (Figure 4F), supporting enhancement of photorespiratory metabolite turnover. Total organic acids were elevated in GDC overexpression lines during the night and under standard light, but this difference diminished under prolonged high light, remaining significant only at early time points (Figure 4G), indicating dynamic rebalancing of central metabolism during acclimation.

Together, these results demonstrate that optimized photorespiration not only supports increased photosynthetic performance but also drives a dynamic reprogramming of carbon allocation under high light, favouring the accumulation of sucrose and starch over amino acids. These effects are particularly present in the double overexpression line H18L2, consistent with a synergistic enhancement of metabolic flexibility under conditions of elevated photorespiratory pressure.

### 3.8 GDC Overexpression Maintains Elevated Rubisco Capacity During Short‐Term High‐Light Exposure

Given the enhanced photosynthetic performance under high light (Figures 1 and S2), reduced O_2_ sensitivity (Figure 2), and the dynamic reprogramming of carbon allocation during short‐term high‐light exposure (Figure 4), we next assessed whether these changes are associated with alterations in Rubisco activity. This was a further relevance as the carboxylation capacity has been suggested to represent a major limitation of CO_2_ assimilation under different environmental conditions (Paillassa et al. 2024). Under growth light conditions (150 µmol m^−2^ s^−1^, 12/12 h photoperiod, 12 h illumination), Rubisco activity showed a tendency toward higher values in the single GDC overexpression lines (Table 2). However, this increase was statistically significant only in PsL2 for both initial (*V*_i_) and total activity (*V*_t_). The result somewhat differs from our previous report, showing only minor effects on Rubisco activity (Timm et al. 2015), but might well be explained due to higher growth light intensity, a longer photoperiod, and a later sampling timepoint during the day. Following exposure to high light (500 µmol m^−2^ s^−1^), these differences became more pronounced. Total Rubisco activity (*V*_t_) was consistently and significantly increased in all GDC overexpression lines, whereas increases in initial activity (*V*_i_) remained restricted to PsL2. Consequently, Rubisco activation state was not significantly altered under either condition studied here. Notably, Rubisco *V*_t_ (all GDC overexpressors) and total soluble protein content increased in FpH18 and H18L2 after high‐light treatment (Table 2), indicating an enhanced capacity for CO_2_ fixation. Together, these results demonstrate that GDC overexpression facilitates increased Rubisco capacity especially during high‐light exposure, which likely contributes to sustained photosynthetic performance and efficient carbon assimilation under conditions of elevated photorespiratory demand.

**Table 2:** Rubisco parameters in GDC‐modulated lines and the wild type. Plants were grown in normal air under environmental controlled conditions (square light – 150 µmol m^−2^ s^−1^) to growth stage 5.1 (Boyes et al. 2001) and then exposed to high light (500 µmol m^−2^ s^−1^). Rubisco activities were determined under both conditions and are given are means ± SD (*n* = 5; independent biological replicates). Values marked with an asterisk are significantly different from the control as determined by Student's *t*‐test (*p* < 0.05).

Genotype	Initial activity (*V*_i_, µmol m^−2^ s^−1^)	Total activity (*V*_t_, µmol m^−2^ s^−1^)	Activation state (%)	Total soluble protein (g m^−2^)
Parameter (square light—150 µmol m^−2^ s^−1^)				
Col.0	13.93 ± 2.29	17.89 ± 2.79	0.78 ± 0.10	3.01 ± 0.43
FpH18	20.78 ± 5.50	24.88 ± 6.94	0.84 ± 0.06	3.52 ± 0.91
PsL2	21.27 ± 3.67*	26.59 ± 5.34*	0.81 ± 0.07	4.00 ± 0.65
H18L2	13.86 ± 2.63	16.67 ± 2.82	0.83 ± 0.05	2.93 ± 0.26
Parameter (fluctuating light—500 µmol m^−2^ s^−1^)				
Col.0	17.53 ± 6.78	24.50 ± 8.24	0.71 ± 0.09	3.91 ± 0.99
FpH18	24.40 ± 5.59	36.61 ± 5.49*	0.66 ± 0.09	5.08 ± 0.48*
PsL‐2	25.65 ± 4.24*	34.57 ± 6.07*	0.75 ± 0.09	4.76 ± 0.63
H18L2	24.37 ± 5.22	35.82 ± 6.97*	0.68 ± 0.04	5.12 ± 0.67*

## 4 Discussion

Photorespiration has long been viewed as a metabolic burden of C_3_ photosynthesis, particularly in the context of crop yield (Busch 2020; Smith et al. 2025). Recent work has demonstrated that targeted manipulation of this pathway can enhance CO_2_ assimilation and plant growth. Two principal strategies emerged: the introduction of synthetic bypasses to reduce CO_2_ release and energetic costs of photorespiration (Kebeish et al. 2007; South et al. 2019; Chen et al. 2026), or the elevated efficiency of the native pathway by improving the activity of specific enzymatic steps to decrease critical metabolite levels and relieve negative feedback regulation on photosynthesis (Timm et al. 2012, 2015, 2025; Flügel et al. 2017; Smith et al. 2024). Despite the success of both approaches, the mechanistic basis underlying increased growth and photosynthetic performance remains partially resolved. However, a unifying mechanism may involve negative feedback regulation exerted by inhibitory metabolite pools within the pathway. Synthetic bypasses initiate glycolate metabolism in the chloroplast, which is proposed to increase local CO_2_ concentrations and and to suppress Rubisco oxygenation. This is expected to also reduce 2PG formation and limit the formation and turnover of downstream photorespiratory intermediates, simultaneously lowering energetic costs. This hypothesis is consistent with measured metabolite profiles in several photorespiration bypass mutant lines (Kebeish et al. 2007; Maier et al. 2012; Dalal et al. 2015; Shen et al. 2019; South et al. 2019; Chen et al. 2024). Conversely, optimizing the activity of key native enzymes, such as PGLP1 and GDC, eventually accelerates photorespiratory metabolism and increases the overall pathway capacity. Despite their different implementations, both strategies may converge on a common outcome: alleviation of metabolic constraints on the CBBC, that is, RuBP regeneration, and improved CO_2_ assimilation.

Our study reveals that upregulation of GDC components is associated with reduced steady‐state levels of photorespiratory intermediates and improved photosynthetic performance, particularly under conditions that promote photorespiration (Figures 1, 2 and 4; S2). Systematic comparison of single and double overexpression lines revealed that simultaneous upregulation of the GDC H‐ and l‐proteins resulted in similar growth responses but stronger physiological and metabolic effects than individual overexpression, consistent with a synergistic enhancement of pathway efficiency (Figures 1, 2, 3, 4). This is evident at multiple levels, including increased electron transport and CO_2_ assimilation under high light, reduced O_2_ sensitivity of photosynthesis, and sustained Rubisco capacity during short‐term high‐light exposure (Figures S2, 1 and 2; Table 2). At the metabolic level, GDC overexpression is associated with a reconfiguration of central carbon metabolism, including reduced steady‐state photorespiratory and CBBC metabolite pools, and a shift in carbon allocation toward soluble sugars and starch, particularly under high light conditions (Figures 3 and 4; Tables 1 and S6–S8). These changes occur without major alterations in stomatal morphology (Table 1), indicating that enhanced carbon assimilation is primarily driven by metabolic rather than structural adjustments observed with GC‐specific PGLP1 manipulation (Sun et al. 2026). One possible contributing factor is higher carbon and energy availability in GCs, as suggested by elevated GC starch levels, which have been linked to efficient stomatal dynamics (Flütsch et al. 2020). Together, these findings are consistent with a model suggesting optimized photorespiration alleviates metabolic constraints on photosynthetic components, thereby enhancing metabolism and more efficient acclimation to conditions imposing high photorespiratory demand (Walker et al. 2024; Walker and Schroeder 2026). The data presented in this manuscript, together with findings from previous studies on leaf‐specific PGLP1 and GDC manipulations (Flügel et al. 2017; Timm et al. 2012, 2015), support a model in which alleviation of the negative feedback exerted by photorespiratory intermediates on the CBBC enhances its overall performance (Figure 5). One possible consequence of improved CBBC performance is increased carbon sink capacity, increasing the CO_2_ demand of mesophyll cells, in turn promoting higher stomatal conductance (Figures 1 and 2). Enhanced CBBC performance is further reflected in the GDC overexpressors, where increased carbon export from the cycle is associated with increased sucrose and starch accumulation (Figure 4). Although the light reactions also responded to GDC manipulation, suggesting an accelerated photosynthetic electron transport rate driven by increased NADPH consumption within the CBBC, this effect is likely secondary under high irradiance, as it was not observed under growth‐light conditions (Figure S2).

**Figure 5:**
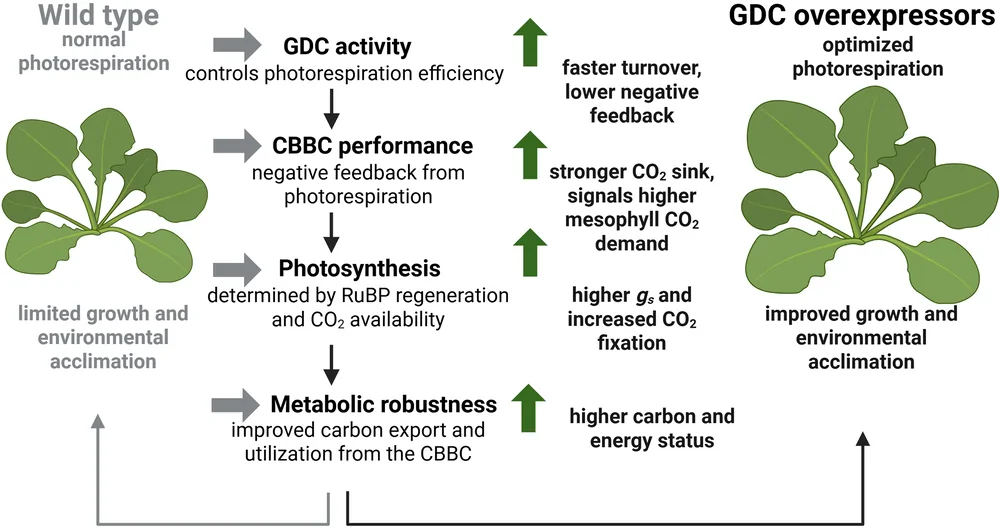
Optimized photorespiration and its effects. Wild‐type photorespiration efficiency is determined by the GDC activity, which exerts control over the turnover of critical pathway intermediates (e.g., glycine, glycolate and 2PG; summarized in Timm and Hagemann 2020), in turn limiting CBBC performance and photosynthetic CO_2_ fixation. GDC‐optimized *Arabidopsis* plants are characterized by improved metabolism of photorespiratory intermediates, which leads to alleviation of negative feedback onto the CBBC operation. Consequently, carbon export and downstream utilization is improved, leading to an increased CO_2_ sink signal in the mesophyll, potentially contributing to increased stomatal opening. The enhanced cellular carbon and energy status supports metabolic robustness and environmental acclimation capacity. GDC, glycine decarboxylase. [Color figure can be viewed at wileyonlinelibrary.com]

The observed synergistic effects of combined GDC‐H and GDC‐L overexpression likely reflect the multienzyme nature and tight coordination of GDC. GDC operates as a hetero‐multimeric enzyme system composed of P‐, H‐, T‐, and l‐proteins, in which efficient glycine decarboxylation depends on the balanced interaction of all components (Douce et al. 2001; Heineke et al. 2001; Bykova et al. 2005, 2014). While increasing individual subunits can enhance pathway activity to some extent (Timm et al. 2012, 2015; López‐Calcagno et al. 2019), imbalances may also arise if other components or cofactors become limiting (López‐Calcagno et al. 2019). In this context, simultaneous upregulation of H‐ and l‐proteins is expected to improve the coordination of catalytic reactions within the complex. Consistent with this interpretation, the double overexpression line exhibited more pronounced metabolic and physiological responses under high light and elevated O_2_ conditions (Figures 1, 2, 3, 4). These findings suggest that balanced upregulation of multiple GDC components may be required to maximize the benefit of photorespiratory capacity enhancement, but only at defined stoichiometric relationships, also involving the GDC subunits P and T. Future work should address this question at higher resolution and test for further improvements in triple and quadruple GDC overexpression lines. The results further highlight the importance of considering multienzyme stoichiometry in metabolic engineering strategies, alongside with other pathway engineering approaches (Simkin et al. 2015; López‐Calcagno et al. 2019). Further, GDC‐L functioning is not restricted to photorespiration but also serves in other key mitochondrial complexes of the tricarboxylic acid cycle and the degradation of branched chain amino acids (discussed in detail before; Timm and Hagemann 2020). Given the role of GDC‐L in pyruvate dehydrogenase, 2‐oxoglutarate dehydrogenase and branched‐chain α‐ketoacid dehydrogenase complexes, some of the observed metabolic responses may additionally reflect effects beyond photorespiratory GDC.

Our data support a model based on a central role of metabolite‐mediated feedback regulation linking photorespiration to photosynthesis (Flügel et al. 2017; Fernie and Bauwe 2020). Reduced steady‐state levels of key intermediates, including 2PG, glycine, and glycerate, are consistent with enhanced photorespiratory metabolism. The consistent decrease in the Gly/Ser ratio, widely used as proxy for the pathway activity (Wingler et al. 2000; Timm et al. 2013; Fu et al. 2023), supports this interpretation (Figure 5). Lower 2PG levels are particularly relevant, as this metabolite is known to directly inhibit several enzymes of the CBBC and associated pathways, including triose phosphate isomerase, SBPase, and phosphofructokinase (Anderson 1971; Flügel et al. 2017; Kelly and Latzko 1976; Li et al. 2019). Its reduction may therefore relieve constraints on both carbon fixation and downstream carbon metabolism (Figure 5). Consistent with this view, GDC overexpression was associated with altered CBBC metabolite profiles, including changes in pentose phosphates, RuBP, and 3PGA, suggesting altered CBBC metabolite homeostasis rather than isolated changes in individual metabolite pools (Figure 4 and Table S6). While isotope labelling approaches will be required to directly quantify metabolic fluxes, the combined physiological and metabolic data support a model in which improved conversion of photorespiratory intermediates reduces feedback inhibition on the CBBC, RuBP regeneration, and promotes more efficient carbon assimilation and export.

In contrast to photorespiratory bypass strategies, which aim to reduce carbon and energy losses by rerouting glycolate metabolism, our results support the concept that optimizing endogenous photorespiratory components represent an effective alternative for improving photosynthetic performance. Synthetic bypasses have demonstrated substantial gains in biomass production under controlled and field conditions (South et al. 2019; Meacham‐Hensold et al. 2024), primarily by minimizing CO_2_ release and potentially increasing chloroplastic CO_2_ availability. However, such approaches have in some cases been associated with altered (i.e., increased) levels of photorespiratory intermediates, which may affect interactions with nitrogen metabolism, redox balance, or metabolic signaling (Eisenhut et al. 2017; Busch 2020; Walker and Schroeder 2026). In contrast, increasing metabolic turnover through the native pathway via coordinated GDC upregulation improves photosynthetic performance without apparent disruption of the photorespiratory nitrogen cycle, largely unchanged organic acid pools, and the absence of detrimental phenotypes (Figures 1, 3, and 4). The stable ammonia levels further suggest that nitrogen re‐assimilation via the GS/GOGAT cycle was not limiting under the conditions tested. Rather than bypassing photorespiration in mutant backgrounds with partially impaired metabolite influx (South et al. 2019; Meacham‐Hensold et al. 2024), the endogenous optimization strategy appears to alleviate constraints within the pathway, thereby improving its integration with central carbon and nitrogen metabolism and enabling more efficient carbon utilization under conditions of elevated photorespiratory pressure.

The physiological and metabolic responses observed in GDC overexpression lines support the importance of photorespiratory capacity for plant performance under dynamic environmental conditions. Under short‐term high light, transgenic lines maintained metabolic robustness. Elevated accumulation of soluble sugars and starch, together with reduced 3PGA levels, suggests improved carbon allocation toward storage and export pathways. These effects were pronounced under high light conditions, pointing to a higher capacity for metabolic adjustment when photosynthetic demand is elevated (Figure 4 and Table S7). The observed metabolic changes further support the complex integration of photorespiration with central metabolic pathways, including glycolysis and the tricarboxylic acid cycle, although the exact flux distributions remain to be resolved. The shift in carbon partitioning under high light was accompanied by increased total soluble protein levels in some lines (Figure 4 and Table 2), suggesting enhanced protein synthesis capacity, particularly in photosynthetic proteins. These observations further suggest that enhanced photorespiratory capacity contributes to metabolic robustness and flexible carbon allocation, key traits for acclimation to fluctuating light and temperature conditions (Zandalinas et al. 2022; Bulut et al. 2025). They also support the concept that the photorespiratory cycle fulfills various important functions, such as N‐assimilation, high‐light and drought acclimation, amino acid biosynthesis and metabolic control, C1 metabolism and, very recently, epigenetic regulation via DNA methylation (Foyer et al. 2009; Busch 2020; Xi et al. 2025; Hankofer et al. 2026). In accordance with previous studies, our findings strengthen the view that improving endogenous photorespiratory capacity represents a promising strategy for enhancing photosynthetic performance while maintaining integration with primary metabolism.

Despite these promising results, potential limitations and trade‐offs of enhanced photorespiratory capacity should be considered. The benefits observed here are likely context‐dependent and may diminish under conditions of low photorespiratory requirements, such as elevated atmospheric CO_2_. Moreover, increasing pathway capacity could impose additional energetic or reductant demands, which might become limiting under certain environmental or developmental conditions. While our data are consistent with a role for reduced metabolite‐mediated feedback inhibition, direct flux measurements will be necessary to quantify pathway dynamics and identify potential regulatory nodes. While no major negative effects were detected in this study, a comprehensive assessment of long‐term performance and potential trade‐offs under field conditions will be required to fully evaluate the robustness of this strategy. Future studies should also explore the integration of photorespiratory optimization with other metabolic engineering strategies to assess potential synergistic effects on photosynthesis and growth.

## 5 Conclusion

Coordinated optimization of GDC activity shapes photorespiratory efficiency and promotes photosynthetic robustness, metabolic flexibility, and improved carbon allocation, particularly under conditions of high photorespiratory pressure. The synergistic effects observed in the GDC‐H and GDC‐L double overexpression line underscore the importance of balanced optimization of multicomponent enzyme systems. These findings establish endogenous photorespiration as a promising target for improving plant performance and abiotic stress resistance under changing environments and suggest that combining pathway optimization with complementary engineering strategies may provide further gains in photosynthetic efficiency and crop productivity.

## Conflicts of Interest

The Arabidopsis Genome Initiative or GenBank/EMBL database contains sequence data from this article under the following accession numbers: *Flaveria pringlei* GDC‐H (FpH; Z25856), GDC‐T (At1g11680), GDC‐P1 (At4g33010), GDC‐H3 (At1g32470), GDC‐L1 (mtLPD1) (At1g48030), SHMT1 (At4g37930), PGLP1 (At5g35700), RbcL (ATCG00490), 40S ribosomal protein S16 (At2g09990), pea LPD (PsL; P31023).

## Supporting information


**Figure S1.** Genotypic verification of H18L2 and protein expression in the GDC overexpression lines and the wild type.
**Figure S2.** Chlorophyll fluorescence parameters of GDC overexpression lines and the wild type.
**Table S1.** Quantitative growth data of GDC overexpression lines and the wild type in different photoperiods.
**Table S2.** Numeric data of chlorophyll fluorescence measurements of GDC overexpression lines and the wild type.
**Table S3.** Numeric data of light response curves of GDC overexpression lines and the wild type.
**Table S4.** Numeric data of O_2_‐dependent CO_2_ response curves of GDC overexpression lines and the wild type.
**Table S5.** Calculated parameters from the O_2_‐dependent CO_2_ response curves of GDC overexpression lines and the wild type.
**Table S6.** Absolute metabolite abundances in GDC overexpressors compared to wild type.
**Table S7.** Absolute metabolite abundances in GDC overexpressors compared to wild type in normal light and high light.

## Data Availability

The data that support the findings of this study are available from the corresponding author upon reasonable request.
