# Supplementary material for: Multi‐Subunit Engineering of Photorespiratory Glycine Decarboxylase Refines Photosynthesis, Growth and Metabolic Robustness of Arabidopsis
Source: Plant Cell Environ. 2026 Aug 21;49(12):9571–85. doi: 10.1111/pce.70836 (PMC13725254; doi:10.1111/pce.70836)
Supplement: Supplementary file 1 [file PCE-49-9571-s001.docx]

**Supporting Information**

**Supporting Figures**

**Supplemental Figure S1.** **Genotypic verification and protein expression in the double GDC overexpressor H18L2.**

**(A)** PCR verification of diagnostic fragments for the *GDC-L* (1902 bp) and *GDC-H* (885 bp) overexpression constructs in six independent individuals of the double *GDC* overexpressor line (H18L2) and the wildtype (WT). The *S16* gene (432 bp) was amplified to verify DNA-integrity. **(B)** Immunoblots of GDC-L (54.00 kDa) and GDC-H (15.23 kDa) expression in the same six independent H18L2 individuals. Signals of mitochondrial SHMT1 (57.40 kDa) were detected to verify equal loading of the proteins. All genotypes were grown under standard conditions to stage 5.1 (Boyes et al., 2001) and leaf-material harvested and analyzed at middle of the light period (6 h illumination).

**Supplemental Figure S2. Genotypic verification of H18L2 and protein expression in the GDC overexpression lines and the wildtype.**

Displayed are selected parameters associated with PSII and PSI integrity and functioning of all genotypes grown under standard growth conditions to stage 5.1 (Boyes et al., 2001). Given are: **(A)** Maximum efficiency of PSII (*F_v_*/*F_m_*) and maximum oxidizable P700 (*P_m_*) from dark adapted plants; **(B)** photosynthetic efficiency curves of PSII (Y[II]) and PSI (Y[I]); **(C)** relative electron transport rates of PSII (rETR II) and PSI (rETR I); **(D)** non-photochemical quenching of PSII (NPQ) and cyclic electron flow around PSI (CET); and, **(E)** acceptor (Y[NA]) and donor (Y[ND]) side limitation of PSI. Shown are means ± SD of at least 6 biological replicates. Values that do not share the same letter are significantly different from each other as determined by ANOVA. Note, statistical evaluation of B to E is given in Supplementary Tab. S2 to facilitate readability.

**Supplemental Tables**

**Supplemental Table S1.** Quantitative growth data of GDC overexpression lines and the wild type grown in different photoperiods.

Plants were grown under standard conditions in normal air in three different photoperiods to stage 5.1 (Boyes et al., 2001). Selected quantitative growth data are given short day (10/14 h day/night-cycle), mid-day (12/12 h day/night-cycle) and long day (14/10 h day/night-cycle), respectively. Given are means ± SD of 8-12 biological replicates. Values that do not share the same letter (bold) are signiﬁcantly different from each other as determined by ANOVA.

| **Parameter** | **Genotype** |  |  |  |
| --- | --- | --- | --- | --- |
|  | **Col.0** | **FpH18** | **PsL2** | **H18L2** |
| ***Photoperiod (10/14 h day/night – growth chamber)*** | | | | |
| **Diameter** | 11.76 ± 0.76^a^ | **14.44 ± 1.51^b^** | **13.53 ± 0.49^b^** | **14.74 ± 1.41^b^** |
| **Leaf-count** | 30.50 ± 2.80^a^ | **36.00 ± 2.45^b^** | **37.00 ± 2.62^b^** | **35.90 ± 2.92^b^** |
| **Fresh weight** | 4.03 ± 0.67^a^ | 4.74 ± 0.98^a^ | **5.18 ± 1.04^b^** | **4.87 ± 0.69^b^** |
| **Dry weight** | 0.37 ± 0.06^a^ | 0.41 ± 0.11^a^ | **0.51 ± 0.11^b^** | **0.51 ± 0.19^b^** |
| **FW/DW ratio** | 10.77 ± 0.65^a^ | **11.98 ± 1.87^b^** | 10.32 ± 1.28^a^ | 10.52 ± 3.68^a^ |
| ***Photoperiod (12/12 h day/night – growth chamber)*** | | | | |
| **Diameter** | 10.89 ± 0.94^a^ | **11.97 ± 1.07^b^** | **13.13 ± 1.23^b^** | **13.72 ± 1.50^b^** |
| **Leaf-count** | 15.00 ± 1.68^a^ | **17.80 ± 2.20^b^** | **21.40 ± 1.96^b^** | **19.63 ± 2.31^b^** |
| **Fresh weight** | 2.77 ± 0.56^a^ | **3.43 ± 0.41^b^** | **4.76 ± 0.38^c^** | **4.49 ± 0.66^c^** |
| **Dry weight** | 0.20 ± 0.04^a^ | **0.26 ± 0.03^b^** | **0.42 ± 0.05^c^** | **0.45 ± 0.09^c^** |
| **FW/DW ratio** | 13.67 ± 0.46^a^ | 13.29 ± 0.33^a^ | **11.48 ± 0.69^b^** | **10.16 ± 1.14^b^** |
| ***Photoperiod (14/10 h day/night – glass house)*** | | | | |
| **Diameter** | 8.91 ± 0.88^a^ | **10.16 ± 0.45^b^** | **10.80 ± 0.67^b^** | **10.80 ± 1.16^b^** |
| **Leaf-count** | 14.80 ± 2.65^a^ | **18.40 ± 1.26^b^** | **19.10 ± 2.23^b^** | **17.95 ± 2.21^b^** |
| **Fresh weight** | 0.77 ± 0.16^a^ | **1.21 ± 0.20^b^** | **1.13 ± 0.24^b^** | **1.24 ± 0.23^b^** |
| **Dry weight** | 0.06 ± 0.02^a^ | **0.11 ± 0.02^b^** | **0.11 ± 0.02^b^** | **0.11 ± 0.02^b^** |
| **FW/DW ratio** | 12.80 ± 0.77^a^ | **10.95 ± 0.30^b^** | **10.35 ± 1.05^b^** | **11.73 ± 0.46^b^** |

**Supplemental Table S2.** Numeric data of chlorophyll fluorescence measurements of GDC overexpression lines and the wild type.

Plants were grown under environmental controlled conditions in air (400 ppm CO_2_) to growth stage 5.1 (Boyes et al. 2001). Light response curves were measured from low-to-high light (34, 62, 141, 237, 489, 756, 1144, and 1757 μmol m^-2^ s^-1^) following 10 min of dark adaptation. Shown are: photosynthetic efficiency of PSII (Y[II]) and PSI (Y[I]), relative electron transport rates of PSII (rETR II) and PSI (rETR I), non-photochemical quenching of PSI (NPQ), cyclic electron transport around PSI (CET) and acceptor (Y[NA]) and donor (Y[ND]) side limitation of PSI parameters. Values are means ± SD (n = 6). Values that do not share the same letter (in bold) are significantly different from each other as determined by ANOVA.

|  |  | **Light intensity (μmol m^-2^ s^-1^)** | | | | | | | |
| --- | --- | --- | --- | --- | --- | --- | --- | --- | --- |
|  |  | **34** | **62** | **86** | **141** | **237** | **489** | **756** | **1144** |
| Y(II) | Col.0 | 0.70 ± 0.02^a^ | 0.73 ± 0.01^a^ | 0.74 ± 0.01^a^ | 0.74 ± 0.01^a^ | 0.73 ± 0.01^a^ | 0.60 ± 0.06^a^ | 0.43 ± 0.05^a^ | 0.27 ± 0.03^a^ |
|  | FpH18 | 0.70 ± 0.03^a^ | 0.73 ± 0.02^a^ | 0.74 ± 0.01^a^ | 0.74 ± 0.01^a^ | 0.73 ± 0.02^a^ | 0.61 ± 0.04^a^ | 0.45 ± 0.03^a^ | 0.29 ± 0.02^a^ |
|  | PsL2 | 0.71 ± 0.03^a^ | 0.74 ± 0.02^ab^ | 0.75 ± 0.01^ab^ | 0.75 ± 0.01^a^ | 0.74 ± 0.02^a^ | 0.63 ± 0.02^a^ | 0.46 ± 0.03^a^ | 0.30 ± 0.02^a^ |
|  | H18L2 | 0.73 ± 0.02^a^ | 0.75 ± 0.01^b^ | 0.76 ± 0.01^b^ | 0.75 ± 0.01^a^ | 0.74 ± 0.01^a^ | **0.67 ± 0.01^b^** | **0.52 ± 0.03^b^** | **0.34 ± 0.03^b^** |
| Y(I) | Col.0 | 0.58 ± 0.02^a^ | 0.65 ± 0.02^a^ | 0.69 ± 0.02^a^ | 0.70 ± 0.03^a^ | 0.72 ± 0.03^a^ | 0.68 ± 0.04^a^ | 0.54 ± 0.04^a^ | 0.38 ± 0.03^a^ |
|  | FpH18 | 0.61 ± 0.03^a^ | 0.67 ± 0.02^a^ | 0.71 ± 0.03^a^ | 0.73 ± 0.02^a^ | 0.74 ± 0.03^a^ | 0.68 ± 0.03^a^ | 0.56 ± 0.02^a^ | 0.41 ± 0.05^a^ |
|  | PsL2 | 0.57 ± 0.03^a^ | 0.65 ± 0.02^a^ | 0.70 ± 0.02^a^ | 0.71 ± 0.02^a^ | 0.71 ± 0.02^a^ | 0.70 ± 0.02^a^ | 0.58 ± 0.03^ab^ | 0.41 ± 0.02^a^ |
|  | H18L2 | 0.60 ± 0.01^a^ | 0.67 ± 0.01^a^ | 0.70 ± 0.01^a^ | 0.71 ± 0.01^a^ | 0.72 ± 0.01^a^ | 0.69 ± 0.01^a^ | **0.61 ± 0.01^b^** | **0.45 ± 0.01^b^** |
| rETR (II) | Col.0 | 10.0 ± 0.3^a^ | 19.0 ± 0.3^a^ | 26.7 ± 0.2^a^ | 43.7 ± 0.2^a^ | 72.3 ± 0.9^a^ | 122.1 ± 11.4^a^ | 135.0 ± 14.5^a^ | 130.3 ± 12.8^a^ |
|  | FpH18 | 10.1 ± 0.5^ab^ | 19.1 ± 0.5^ab^ | 26.8 ± 0.4^ab^ | 43.8 ± 0.7^ab^ | 72.4 ± 2.0^ab^ | 125.6 ± 7.3^a^ | 142.4 ± 8.7^a^ | 141.3 ± 9.6^ab^ |
|  | PsL2 | 10.2 ± 0.4^ab^ | 19.3 ± 0.4^ab^ | 27.0 ± 0.4^ab^ | **44.3 ± 0.3^b^** | 73.6 ± 0.3^ab^ | 129.5 ± 4.1^a^ | 146.6± 9.6^a^ | 144.3± 8.3^ab^ |
|  | H18L2 | **10.5 ± 0.2^b^** | **19.6 ± 0.3^b^** | **27.3 ± 0.2^b^** | **44.6 ± 0.3^b^** | **74.0 ± 0.3^b^** | **138.1 ± 2.9^b^** | **164.0 ± 9.9^b^** | **163.9 ± 16.5^b^** |
| rETR (I) | Col.0 | 8.2 ± 0.3^a^ | 16.9 ± 0.4^a^ | 25.1± 0.6^a^ | 41.6 ± 1.7^a^ | 71.8 ± 3.4^a^ | 140.0 ± 7.8^a^ | 171.5 ± 12.5^a^ | 180.1 ± 12.9^a^ |
|  | FpH18 | 8.7 ± 0.4^a^ | 17.5 ± 0.5^a^ | 25.7 ± 1.0^a^ | 43.1 ± 1.5^a^ | 73.4 ± 3.0^a^ | 139.5 ± 6.6^a^ | 176.3 ± 7.2^a^ | 197.0 ± 21.7^ab^ |
|  | PsL2 | 8.2 ± 0.4^a^ | 17.0 ± 0.5^a^ | 25.1 ± 0.8^a^ | 42.2 ± 1.1^a^ | 70.7 ± 1.5^a^ | 143.2 ± 3.6^a^ | 183.9 ± 8.6^ab^ | 196.3 ± 10.6^ab^ |
|  | H18L2 | 8.6 ± 0.2^a^ | 17.3 ± 0.5^a^ | 25.4 ± 0.2^a^ | 42.2 ± 0.8^a^ | 71.4 ± 1.4^a^ | 142.4 ± 3.0^a^ | **194.0 ± 6.1^b^** | **218.1 ± 18.8^b^** |
| NPQ | Col.0 | 0.09 ± 0.03^a^ | 0.10 ± 0.04^a^ | 0.14 ± 0.04^a^ | 0.17 ± 0.05^a^ | 0.20 ± 0.05^a^ | 0.53 ± 0.02^a^ | 1.03 ± 0.02^a^ | 1.35 ± 0.01^a^ |
|  | FpH18 | 0.11 ± 0.06^a^ | 0.10 ± 0.02^a^ | 0.14 ± 0.03^a^ | 0.17 ± 0.03^a^ | 0.19 ± 0.04^a^ | 0.43 ± 0.08^b^ | 0.90 ± 0.12^b^ | 1.22 ± 0.11^b^ |
|  | PsL2 | 0.07 ± 0.03^a^ | 0.10 ± 0.02^a^ | 0.13 ± 0.03^a^ | 0.17 ± 0.04^a^ | 0.19 ± 0.04^a^ | 0.41 ± 0.07^b^ | 0.93 ± 0.10^b^ | 1.29 ± 0.07^b^ |
|  | H18L2 | 0.05 ± 0.03^a^ | 0.08 ± 0.03^a^ | 0.12 ± 0.04^a^ | 0.15 ± 0.05^a^ | 0.17 ± 0.05^a^ | 0.29 ± 0.07^b^ | 0.76 ± 0.12^b^ | 1.15 ± 0.11^b^ |
| CET | Col.0 | - 1.8 ± 0.2^a^ | - 2.1 ± 0.4^a^ | - 1.6 ± 0.5^a^ | - 2.1 ± 1.6^a^ | - 0.5 ± 2.7^a^ | 17.8 ± 5.3^a^ | 36.5 ± 5.5^a^ | 49.8 ± 3.5^a^ |
|  | FpH18 | - 1.4 ± 0.5^a^ | - 1.6 ± 0.4^a^ | - 1.0 ± 0.7^a^ | - 0.7 ± 1.1^a^ | 1.0 ± 1.9^a^ | 13.9 ± 4.8^a^ | 33.9 ± 9.7^a^ | 55.6 ± 9.2^a^ |
|  | PsL2 | - 2.0 ± 0.4^a^ | - 2.3 ± 0.5^a^ | - 1.9 ± 0.6^a^ | - 2.2 ± 1.1^a^ | - 2.9 ± 1.7^a^ | 13.7 ±2.3^a^ | 37.3 ± 3.2^a^ | 52.0 ± 4.1^a^ |
|  | H18L2 | - 1.8 ± 0.2^a^ | - 2.3 ± 0.4^a^ | - 1.9 ± 0.7^a^ | - 2.5 ± 1.4^a^ | - 2.5 ± 3.1^a^ | 4.3 ± 7.9^a^ | 30.0 ± 7.7^a^ | 54.2 ± 6.1^a^ |
| Y(NA) | Col.0 | 0.42 ± 0.02^a^ | 0.34 ± 0.02^a^ | 0.29 ± 0.02^a^ | 0.28 ± 0.03^a^ | 0.25 ± 0.03^a^ | 0.13 ± 0.02^a^ | 0.08 ± 0.02^a^ | 0.08 ± 0.02^a^ |
|  | FpH18 | 0.39 ± 0.03^a^ | 0.32 ± 0.02^a^ | 0.28 ± 0.03^a^ | 0.26 ± 0.02^a^ | 0.23 ± 0.03^a^ | 0.17 ± 0.06^a^ | 0.12 ± 0.05^a^ | 0.10 ± 0.04^a^ |
|  | PsL2 | 0.42 ± 0.03^a^ | 0.34 ± 0.02^a^ | 0.29 ± 0.02^a^ | 027 ± 0.02^a^ | 0.26 ± 0.02^a^ | 0.16 ± 0.01^a^ | 0.09 ± 0.01^a^ | 0.09 ± 0.01^a^ |
|  | H18L2 | 0.39 ± 0.02^a^ | 0.33 ± 0.02^a^ | 0.29 ± 0.02^a^ | 0.27 ± 0.03^a^ | 0.26 ± 0.03^a^ | 0.21 ± 0.05^a^ | 0.13 ± 0.04^a^ | 0.10 ± 0.03^a^ |
| Y(ND) | Col.0 | 0.01 ± 0.02^a^ | 0.01 ± 0.02^a^ | 0.01 ± 0.02^a^ | 0.02 ± 0.01^a^ | 0.03 ± 0.01^a^ | 0.19 ± 0.05^a^ | 0.38 ± 0.04^a^ | 0.55 ± 0.03^a^ |
|  | FpH18 | 0.01 ± 0.01^a^ | 0.01 ± 0.01^a^ | 0.01 ± 0.01^a^ | 0.01 ± 0.01^a^ | 0.03 ± 0.01^a^ | 0.15 ± 0.04^a^ | 0.33 ± 0.07^ab^ | 0.49 ± 0.08^ab^ |
|  | PsL2 | 0.01 ± 0.01^a^ | 0.01 ± 0.01^a^ | 0.01 ± 0.01^a^ | 0.02 ± 0.01^a^ | 0.03 ± 0.01^a^ | 0.15 ± 0.02^a^ | 0.33 ± 0.03^ab^ | 0.51 ± 0.02^ab^ |
|  | H18L2 | 0.01 ± 0.01^a^ | 0.01 ± 0.01^a^ | 0.01 ± 0.01^a^ | 0.02 ± 0.01^a^ | 0.02 ± 0.01^a^ | **0.09 ± 0.02^b^** | **0.26 ± 0.05^b^** | **0.45 ± 0.06^b^** |

**Supplemental Table S3.** Numeric data of light response curves of GDC overexpression lines and the wild type.

Plants were grown under environmental controlled conditions in air (400 ppm CO_2_) to growth stage 5.1 (Boyes et al. 2001). Light response curves were measured by gas exchange from high-to-low light (1600, 1200, 800, 400, 200, 100, 50, 25, and 0 μmol m^-2^ s^-1^) following 10 min of light adaptation at 1000 μmol m^-2^ s^-1^. Given are: net CO_2_ assimilation rate (*A_N_*, μmol m^-2^ s^-1^), stomatal conductance (*g_s_*, mol m^-2^ s^-1^), intracellular CO_2_ concentration (*C_i_*, μmol mol^-1^), transpiration rate (*E*, μmol H_2_O m^-2^ s^-1^); and intrinsic water use efficiency (*WUE*, μmol CO_2_ mol^-2^ H_2_O). Values are means ± SD (n = 6). Values that do not share the same letter (in bold) are significantly different from each other as determined by ANOVA.

|  |  | **Light intensity (μmol m^-2^ s^-1^)** | | | | | | | | |
| --- | --- | --- | --- | --- | --- | --- | --- | --- | --- | --- |
|  |  | **0** | **25** | **50** | **100** | **200** | **400** | **800** | **1200** | **1600** |
|  | Col.0 | - 0.58 ± 0.27^a^ | 0.97 ± 0.41^a^ | 2.51 ± 0.62^a^ | 3.31 ± 0.71^a^ | 4.58 ± 0.52^a^ | 5.46 ± 0.46^a^ | 6.14 ± 0.55^a^ | 6.42 ± 0.64^a^ | 7.31 ± 0.60^a^ |
| ***A*_N_** | FpH18 | - 0.78 ± 0.21^a^ | 1.41 ± 0.31^a^ | 2.91 ± 0.16^a^ | 3.87 ± 0.43^a^ | 5.19 ± 0.50^ab^ | **6.15 ± 0.21^b^** | **7.06 ± 0.21^b^** | **7.75 ± 0.66^b^** | **8.13 ± 0.24^b^** |
|  | PsL2 | - 0.59 ± 0.28^a^ | 1.42 ± 0.43^ab^ | 2.84 ± 0.18^a^ | 3.11 ± 0.22^a^ | 5.44 ± 0.45^ab^ | **6.61 ± 0.66^bc^** | **7.53 ± 0.42^b^** | **8.37 ± 0.45^b^** | **8.69 ± 0.48^b^** |
|  | H18L2 | - 1.10 ± 0.37^a^ | **1.90 ± 0.16^b^** | 2.70 ± 0.33^a^ | 3.36 ± 0.44^a^ | **5.82 ± 0.35^b^** | **7.56 ± 0.63^c^** | **8.67 ± 0.50^c^** | **9.26 ± 0.46^c^** | **9.73 ± 0.51^c^** |
|  | Col.0 | 0.08 ± 0.02^a^ | 0.07 ± 0.01^a^ | 0.07 ± 0.01^a^ | 0.07 ± 0.01^a^ | 0.07 ± 0.01^a^ | 0.07 ± 0.01^a^ | 0.08 ± 0.02^a^ | 0.09 ± 0.02^a^ | 0.11 ± 0.02^a^ |
| ***g*_s_** | FpH18 | **0.13 ± 0.03^b^** | **0.11 ± 0.02^b^** | **0.10 ± 0.02^b^** | 0.09 ± 0.02^a^ | **0.09 ± 0.02^b^** | **0.10 ± 0.02^b^** | **0.11 ± 0.01^b^** | **0.12 ± 0.01^b^** | **0.13 ± 0.01^b^** |
|  | PsL2 | **0.21 ± 0.03^c^** | **0.18 ± 0.02^c^** | **0.15 ± 0.01^c^** | **0.13 ± 0.01^b^** | **0.11 ± 0.01^c^** | **0.11 ± 0.01^b^** | **0.12 ± 0.02^b^** | **0.14 ± 0.02^b^** | **0.16 ± 0.02^c^** |
|  | H18L2 | **0.22 ± 0.05^c^** | **0.21 ± 0.05^c^** | **0.19 ± 0.04^c^** | **0.18 ± 0.04^c^** | **0.17 ± 0.04^d^** | **0.17 ± 0.03^c^** | **0.18 ± 0.03^c^** | **0.19 ± 0.02^c^** | **0.20 ± 0.02^d^** |
|  | Col.0 | 403.9 ± 6.7^a^ | 359.5 ± 5.4^a^ | 305.1 ± 12.1^a^ | 279.4 ± 8.9^a^ | 263.7 ± 13.0^a^ | 244.4 ± 18.5^a^ | 243.5 ± 25.5^a^ | 257.1 ± 12.8^a^ | 262.6 ± 11.0^a^ |
| ***C*_i_** | FpH18 | 400.4 ± 3.9^a^ | **372.6 ± 5.9^b^** | **341.3 ± 6.6^b^** | **320.5 ± 8.9^b^** | **295.1 ±9.7^b^** | **279.6 ± 11.6^b^** | **271.7 ± 4.1^b^** | **270.8 ± 0.5^b^** | **280.4 ± 5.9^b^** |
|  | PsL2 | 397.4 ± 4.6^a^ | **378.4 ± 6.2^b^** | **358.0 ± 6.5^c^** | **347.5 ± 20.3^b^** | **306.9 ± 16.6^b^** | **285.1 ± 19.4^b^** | **279.7 ± 18.3^b^** | **279.3 ± 22.3^b^** | **287.4 ± 20.6^b^** |
|  | H18L2 | 402.6 ± 1.2^a^ | **386.4 ± 1.5^c^** | **370.5 ± 2.4^d^** | **365.9 ± 7.5^c^** | **340.3 ± 7.1^c^** | **323.3 ± 8.5^c^** | **315.3 ± 7.0^c^** | **314.9 ± 7.4^c^** | **315.0 ± 10.9_c_** |
|  | Col.0 | 0.98 ± 0.52^a^ | 0.88 ± 0.35^a^ | 0.84 ± 0.25^a^ | 0.82 ± 0.21^a^ | 0.84 ± 0.21^a^ | 0.91 ± 0.24^a^ | 1.04 ± 0.26^a^ | 1.22 ± 0.29^a^ | 1.46 ± 0.31^a^ |
| ***E*** | FpH18 | **2.24 ± 0.31^b^** | **2.00 ± 0.07^b^** | **1.73 ± 0.10^b^** | **1.54 ± 0.21^b^** | **1.46 ± 0.25^b^** | **1.51 ± 0.28^b^** | **1.69 ± 0.27^b^** | **1.87 ± 0.227^b^** | **2.17 ± 0.25^b^** |
|  | PsL2 | **2.89 ± 1.03^b^** | **2.47 ± 0.87^b^** | **2.07 ± 0.65^b^** | **1.75 ± 0.46^b^** | **1.55 ± 0.33^b^** | **1.47 ± 0.21^b^** | **1.58 ± 0.14^b^** | **1.83 ± 0.13^b^** | **2.16 ± 0.15^b^** |
|  | H18L2 | **1.89 ± 0.40^b^** | **1.81 ± 0.42^b^** | **1.73 ± 0.40^b^** | **1.66 ± 0.37^b^** | **1.62 ± 0.23^b^** | **1.60 ± 0.27^b^** | **1.73 ± 0.23^b^** | **1.92 ± 0.18^b^** | **2.19 ± 0.11^b^** |
|  | Col.0 | - 8.67 ± 2.59^a^ | 15.83 ± 6.56^a^ | 38.73 ± 11.5^a^ | 49.91 ± 14.69^a^ | 60.54 ± 11.74^a^ | 68.71 ± 13.67^a^ | 66.82 ± 8.82^a^ | 62.46 ± 8.57^a^ | 62.56 ± 6.77^a^ |
| ***WUE*** | FpH18 | - 6.58 ± 2.51^a^ | 9.99 ± 2.87^ab^ | 28.88 ± 3.51^a^ | 41.42 ± 5.56^a^ | 56.91 ± 6.05^a^ | 65.94 ± 6.83^a^ | 70.14 ± 2.03^a^ | 70.29 ± 0.93^a^ | 63.78 ± 2.90^a^ |
|  | PsL2 | **- 3.16 ± 1.14^b^** | **7.67 ± 3.16^b^** | **19.56 ±2.80^b^** | **25.77 ± 6.58^b^** | 50.27 ± 8.66^a^ | 63.32 ± 11.11^ab^ | 66.06 ± 17.21^ab^ | 65.73 ± 17.17^ab^ | 60.19 ± 16.05^ab^ |
|  | H18L2 | - 4.97 ± 2.61^ab^ | **4.51 ± 2.64_c_** | **14.41 ± 2.68^c^** | **19.40 ± 8.30^b^** | **35.38 ± 8.01^b^** | **46.68 ± 10.13^b^** | **51.06 ± 10.15^b^** | **51.12 ± 10.33^b^** | **50.08 ± 10.4^b^** |

**Supplemental Table S4.** Numeric data of O_2_-dependent CO_2_ response curves of GDC overexpression lines and the wild type.

Plants were grown under environmental controlled conditions in air (400 ppm CO_2_) to growth stage 5.1 (Boyes et al. 2001). CO_2_ response curves (400, 0, 50, 100, 200, 300, 400 ppm CO_2_) were measured following 10 min of light adaptation (1000 μmol m^-2^ s^-1^) at 3, 21 and 40% O_2_. Given are: net CO_2_ assimilation rate (*A_N_*), stomatal conductance (*g_s_*), intracellular CO_2_ concentration (*C_i_*), transpiration rate (*E*), intrinsic water use efficiency (WUE) and net CO_2_ compensation points (*Γ*). Values are means ± SD (n = 6). Values that do not share the same letter are significantly different (in bold) from each other as determined by ANOVA.

|  | **Genotype** | **Parameter** |  |  |  |  |
| --- | --- | --- | --- | --- | --- | --- |
| **O_2_** |  | ***A*_N_ (μmol m^-2^ s^-1^)** | ***g*_s_ (mol m^-2^ s^-1^)** | ***C*_i_ (μmol mol^-1^)** | ***E* (mol H_2_O m^-2^ s^-1^)** | ***Γ* (μL L^-1^)** |
| **3%** | Col.0 | 12.57 ± 1.44^a^ | 0.157 ± 0.080^ab^ | 260.2 ± 37.0^a^ | 4.40 ± 1.16^a^ | 10.52 ± 4.17^a^ |
|  | FpH18 | 12.24 ± 1.80^a^ | 0.177 ± 0.037^a^ | 257.0 ± 24.8^a^ | 4.24 ± 0.73^a^ | 9.60 ± 3.03^a^ |
|  | PsL2 | 12.43 ± 1.32^a^ | 0.228 ± 0.044^ab^ | 285.3 ± 22.1^a^ | 5.21 ± 0.77^a^ | 9.90 ± 4.27^a^ |
|  | H18L2 | 13.13 ± 1.46^a^ | **0.267 ± 0.031^b^** | 297.6 ± 10.1^a^ | 5.19 ± 0.80^a^ | 9.67 ± 2.97^a^ |
| **21%** | Col.0 | 8.70 ± 0.49^b^ | 0.155 ± 0.037^c^ | 286.5 ± 26.1^b^ | 2.88 ± 0.79^b^ | 64.25 ± 2.58^b^ |
|  | FpH18 | **9.85 ± 0.71^c^** | **0.270 ± 0.060^d^** | **323.3 ± 9.7^c^** | **3.40 ± 0.82^c^** | **58.08 ± 2.88^c^** |
|  | PsL2 | **10.13 ± 0.58^c^** | **0.292 ± 0.051^d^** | **326.1 ± 10.2^c^** | **3.80 ± 0.49^c^** | **57.55 ± 2.21^c^** |
|  | H18L2 | **11.71 ± 0.88^d^** | **0.363 ± 0.071^e^** | **328.5 ± 10.7^c^** | **4.40 ± 0.26^d^** | **51.99 ± 4.16^d^** |
| **40%** | Col.0 | 4.82 ± 0.70^e^ | 0.108 ± 0.013^f^ | 305.0 ± 7.0^d^ | 2.99 ± 0.26^e^ | 127.24 ± 6.23^e^ |
|  | FpH18 | **6.28 ± 0.45^f^** | **0.145 ± 0.019^g^** | **309.5 ± 8.9^e^** | **3.63 ± 0.43^f^** | **109.76 ± 3.67^f^** |
|  | PsL2 | **6.59 ± 0.84^f^** | **0.169 ± 0.015^g^** | **310.3 ± 12.3^e^** | **4.05 ± 0.37^f^** | **112.63 ± 7.81^f^** |
|  | H18L2 | **8.44 ± 1.34^g^** | **0.200 ± 0.019^h^** | **312.0 ± 9.8^e^** | **4.40 ± 0.44^f^** | **97.54 ± 6.74^g^** |
|  |  |  |  |  |  |  |

**Supplemental Table S5.** Calculated parameters from the O_2_-dependent CO_2_ response curves of GDC overexpression lines and the wild type.

Estimations of the O_2_-inhibition of *A_N_* and the slope of the *Γ*-versus-O_2_ concentration functions (*γ*). Given are means ± SD (n=6). Values that do not share the same letter (in bold) are significantly different from each other as determined by ANOVA. Full numerical data of the CO_2_-response curves are provided as Supplementary Table S4.

|  | **Parameter** |  |
| --- | --- | --- |
| **Genotype** | **O_2_ inhibition of *A*_N_ (%)** | ***γ* (slope of *Γ* vs O_2_ curves)** |
| **Col.0** | 44.8 ± 5.4^a^ | 3.18 ± 0.18^a^ |
| **FpH18** | **36.0 ± 5.7^b^** | **2.71 ±0.12^b^** |
| **PsL2** | **37.1 ± 4.3^b^** | **2.79 ± 0.24^b^** |
| **H18L2** | **28.1 ± 7.8^c^** | **2.39 ± 0.14^c^** |

**Supplemental Table S6.** Absolute metabolite abundances in GDC overexpressors compared to wildtype

Plants were grown under environmental controlled conditions (12/12 h day/night cycle; 400 ppm CO_2_; 6 weeks) to growth stage 5.1 (Boyes et al. 2001) and leaf-material harvested at the end of the day (11 h illumination). Soluble sugars were determined by GC and amino acids, organic acids and 3PGA by LC-MS/MS. Given metabolites are means ± SD of at least six biological replicates. Single amino and organic acid contents are given in ng/mg DW^-1^. Soluble sugars and the sum parameters of total soluble sugar (TSS), total amino acids (TAA) and total organic acids (TOA) contents are given in µg/mg DW^-1^. Values marked with an asterisk are significantly different from the wildtype as determined by student´s *t*-test (*p* < 0.05).

| **Metabolite** | **Genotype** |  |  |  |
| --- | --- | --- | --- | --- |
|  | **Col.0** | **FpH18** | **PsL2** | **H18L2** |
| ***Sugars (gc analysis; µg * mg DW^-1^)*** | | | | |
| **TSS** | 13.1 ± 0.9 | **15.4 ± 1.9*** | **15.4 ± 1.9*** | **14.7 ± 1.4*** |
| **Sucrose** | 11.2 ± 0.5 | **13.1 ± 1.2*** | **12.0 ± 0.7*** | **12.3 ± 0.6*** |
| **Glucose** | 1.3 ± 0.2 | 1.7 ± 0.7 | 2.3 ± 0.9 | 1.7 ± 0.9 |
| **Fructose** | 0.8 ± 0.5 | 0.5 ± 0.1 | 1.1 ± 0.5 | 0.6 ± 0.2 |
| ***Amino Acids (LC-MS/MS analysis; ng * mg DW^-1^)*** | | | | |
| **TAA** | 20.5 ± 1.9 | **26.3 ± 2.4*** | **25.0 ± 1.1*** | **24.7 ± 2.8*** |
| **Alanine** | 981.3 ± 79.8 | **1570.5 ± 209.3*** | **1315.9 ± 129.2*** | **1328.5 ± 162.9*** |
| **Arginine** | 218.1 ± 78.1 | 235.2 ± 69.0 | 262.3 ± 45.0 | 281.1 ± 50.5 |
| **Asparagine** | 719.2 ± 115.2 | 780.4 ± 156.0 | 836.3 ± 94.9 | 817.2 ± 118.5 |
| **Aspartate** | 3081.7 ± 247.8 | **2668.5 ± 382.7*** | **2526.9 ± 268.6*** | **2459.7 ± 452.8*** |
| **Glutamate** | 4042.0 ± 360.7 | **5466.6 ± 1015.4*** | **5306.5 ± 413.9*** | **5223.0 ± 752.5*** |
| **Glutamine** | 8427.4 ± 1136.6 | **12061.7 ± 2253.8*** | **10470.4 ± 576.2*** | **9902.6 ± 1363.6*** |
| **Glycine** | 233.5 ± 19.6 | **137.1 ± 65.5*** | **163.7 ± 36.1*** | **186.5 ± 34.3*** |
| **Gly/Ser** | 0.32 ± 0.02 | **0.11 ± 0.06*** | **0.14 ± 0.04*** | **0.17 ± 0.03*** |
| **Histidine** | 49.0 ± 8.32 | 55.7 ± 19.3 | 53.6 ± 9.9 | 52.7 ± 6.1 |
| **Isoleucine** | 42.6 ± 7.9 | **63.2 ± 11.5*** | **60.7 ± 5.1*** | **56.7 ± 6.1*** |
| **Leucine** | 25.3 ± 4.6 | **37.8 ± 7.6*** | **39.3 ± 4.8*** | **35.9 ± 4.9*** |
| **Lysine** | 58.7 ± 11.1 | 74.9 ± 18.8 | **74.6 ± 8.5*** | **73.4 ± 9.9*** |
| **Methionine** | 40.6 ± 6.9 | **68.3 ± 16.0*** | **50.8 ± 6.8*** | **48.8 ± 6.9*** |
| **Phenylalanine** | 161.0 ± 23.0 | 138.2 ± 20.6 | 158.7 ± 29.9 | 135.0 ± 31.3 |
| **Proline** | 429.0 ± 103.5 | **962.9 ± 352.7*** | **807.4 ± 220.4*** | **650.5 ± 163.5*** |
| **Serine** | 1034.1 ± 86.2 | **1305.2 ± 269.7*** | **1144.5 ± 74.2*** | **1147.6 ± 99.6*** |
| **Threonine** | 1226.7 ± 134.1 | **1673.5 ± 275.6*** | **1497.2 ± 66.6*** | 1347.1 ± 192.1 |
| **Tryptophan** | 12.4 ± 2.95 | 12.1 ± 3.5 | 13.0 ± 2.0 | 12.6 ± 2.1 |
| **Tyrosine** | 11.6 ± 2.5 | **15.4 ± 3.4*** | **18.1 ± 2.6*** | **15.6 ± 2.8*** |
| **Valine** | 150.0 ± 21.7 | **234.7 ± 42.0*** | **210.9 ± 12.8*** | **188.9 ± 25.4*** |
| ***Organic acids (LC-MS/MS analysis; ng * mg DW^-1^)*** | | | | |
| **TOA** | 247.0 ± 22.2 | 270.6 ± 38.2 | 261.9 ± 29.9 | 264.1 ± 28.0 |
| **2-OG** | 243.4 ± 28.1 | 211.9 ± 21.6 | **159.0 ± 14.4*** | **162.4 ± 25.3*** |
| **Aconitate** | 516.2 ± 102.7 | 641.6 ± 167.5 | 573.5 ± 44.1 | 593.9 ± 102.7 |
| **Citrate** | 40015.7 ± 7458.6 | 43804.8 ± 12699.1 | 42943.1 ± 7378.5 | 44703.7 ± 7791.7 |
| **Fumarate** | 5396.1 ± 1006.4 | **7253.7 ± 1732.7*** | **7627.7 ± 1657.7*** | **7039.2 ± 1485.9*** |
| **GABA** | 34.4 ± 19.9 | 58.9 ± 18.7* | 82.5 ± 26.5* | 52.1 ± 13.5 |
| **Isocitrate** | 6007.9 ± 2248.5 | 6102.0 ± 1998.5 | 6053.3 ± 922.4 | 7189.5 ± 2540.5 |
| **Lactate** | 124.7 ± 29.0 | 139.0 ± 58.1 | 115.7 ± 35.1 | 96.3 ± 21.1 |
| **Malate** | 19470.1 ± 1604.1 | 22475.1 ± 4766.8 | 20444.8 ± 2410.8 | 20444.7 ± 2038.6 |
| **Pyruvate** | 347.1 ± 23.9 | 342.3 ± 75.2 | **285.4 ± 18.0*** | **294.8 ± 43.8*** |
| **Succinate** | 147.8 ± 30.1 | 168.7 ± 60.7 | 155.0 ± 15.4 | 122.2 ± 24.9 |
| ***Photorespiration/Calvin Benson Bassham cycle (LC-MS/MS analysis; ng * mg DW^-1^)*** | | | | |
| **2PG** | 43.4 ± 5.7 | **19.4 ± 5.6*** | **21.0 ± 5.4*** | **29.0 ± 7.0*** |
| **3PGA** | 275.0 ± 64.9 | **119.4 ± 30.1*** | **123.5 ± 23.3*** | **95.4 ± 18.2*** |
|  |  |  |  |  |
| ***Calvin Benson Bassham cycle (LC-MS/MS analysis; pmol * mg DW^-1^)*** | | | | |
| **ADPG** | 3.0 ± 0.7 | **2.1 ± 0.4*** | 2.3 ± 0.4 | **1.2 ± 0.5*** |
| **DHAP** | 81.2 ± 19.1 | **59.1 ± 11.7*** | 60.7 ± 24.9 | **48.9 ± 17.5*** |
| **F6P** | 694.3 ± 228.9 | 667.0 ± 253.9 | 659.7 ± 123.2 | 605.0 ± 98.8 |
| **FBP** | 29.5 ± 11.1 | 29.0 ± 5.7 | 28.3 ± 5.0 | 21.5 ± 3.4 |
| **G1P** | 209.2 ± 41.7 | 168.1 ± 55.5 | 174.6 ± 42.7 | 162.8 ± 30.2 |
| **G6P** | 2115.6 ± 543.2 | 1771.4 ± 571.6 | 1802.3 ± 335.1 | 1689.3 ± 267.0 |
| **Glycerate** | 5237.6 ± 1195.5 | **3469.2 ± 864.8*** | **3839.8 ± 740.7*** | **3199.6 ± 1317.8*** |
| **R5P** | 66.6 ± 15.5 | 51.4 ± 10.2 | 53.1 ± 10.8 | 53.3 ± 12.8 |
| **Ru5P+Xu5P** | 174.6 ± 38.9 | **109.3 ± 27.8*** | **116.8 ± 27.0*** | **114.3 ± 17.4*** |
| **RuBP** | 82.7 ± 13.1 | **47.9 ± 10.3*** | 73.2 ± 12.7 | **41.2 ± 23.4*** |
| **S7P** | 382.3 ± 89.4 | 300.4 ± 63.9 | 344.5 ± 64.5 | **279.8 ± 41.0*** |
| **SBP** | 23.5 ± 5.5 | 19.8 ± 7.7 | 29.9 ± 6.9 | 18.6 ± 5.8 |
| **Shikimate** | 356.8 ± 51.4 | **284.1 ± 53.3*** | 318.5 ± 73.0 | **287.1 ± 33.0*** |
| **UDPG** | 490.5 ± 183.9 | 417.9 ± 109.5 | 472.4 ± 85.9 | 438.7 ± 177.8 |

**Supplemental Table S7.** Absolute metabolite abundances in GDC overexpressors compared to wildtype in normal and high light

Plants were grown under standard conditions (12/12 h day/night cycle; 400 ppm CO_2_; 6 weeks) to stage 5.1 (Boyes et al. 2001) under normal growth light (~120 µmol m^-2^ ^-1^) and transferred to high light (500 µmol m^-2^ ^-1^). Leaf-material was harvested at the end of the night (EoN; 11 h darkness, control) and after 4, 8 and 12 h normal (NL) and high light (HL) illumination. Selected metabolites were determined by spectrophotometric analysis, gas chromatography and LC-MS/MS. Given metabolites are means ± SD of at least six biological replicates. Values marked with an asterisk are significantly different from the wildtype as determined by student´s T-test (*p* < 0.05).

|  |  | **Light intensity (~120 μmol m^-2^ s^-1^)** | | | | | **Light intensity (500 μmol m^-2^ s^-1^)** | | |
| --- | --- | --- | --- | --- | --- | --- | --- | --- | --- |
| **Metabolite** |  | **EoN** | **NL - 4h** | **NL - 8h** | **NL - 12h** | **HL – 4h** | | **HL – 8h** | **HL – 12h** |
|  | Col.0 | 687.7 ± 144.6 | 716.7 ± 52.3 | 715.1 ± 140.0 | 643.5 ± 74.3 | 813.0 ± 96.7 | | 927.4 ± 148.4 | 730.9 ± 106.1 |
| **NH_4_^+^** | FpH18 | 753.1 ± 86.8 | 647.8 ± 45.2* | 685.1 ± 62.1 | 642.5 ± 90.3 | 889.9 ± 143.6 | | 1006.4 ± 164.6 | 787.8 ± 43.1 |
|  | PsL2 | 788.7 ± 70.2 | 582.5 ± 63.4* | 717.5 ± 85.5 | 821.4 ± 111.0* | 745.3 ± 154.8 | | 762.9 ± 163.3 | 733.1 ± 121.1 |
|  | H18L2 | 747.0 ± 98.2 | 688.1 ± 128.5 | 599.1 ± 86.9 | 772.6 ± 144.6 | 713.9 ± 149.3 | | 734.4 ± 91.5* | 685.5 ± 100.6 |
|  | Col.0 | 2455.4 ± 711.4 | 3480.5 ± 479.9 | 4076.1 ± 388.8 | 4839.9 ± 290.6 | 5857.4 ± 427.0 | | 5852.8 ± 2408.4 | 8447.8 ± 798.8 |
| **Sucrose** | FpH18 | 2608.9 ± 298.9 | 3722.4 ± 434.5 | 3917.6 ± 313.7 | 4324.2 ± 247.7 | 6140.3 ± 548.2 | | 6764.9 ± 865.5 | 10261.2 ± 921.7* |
|  | PsL2 | 3446.9 ± 636.1* | 4251.1 ± 510.1* | 4480.3 ± 362.7 | 5719.1 ± 1074.8* | 7062.0 ± 1449.2* | | 7488.3 ± 492.3 | 10997.3 ± 2390.4* |
|  | H18L2 | 3432.3 ± 1003.2 | 4029.9 ± 722.4 | 4147.7 ± 617.7 | 5565.2 ± 899.6* | 6399.2 ± 987.7 | | 7562.8 ± 2397.0 | 11674.7 ± 2773.2* |
|  | Col.0 | 9.8 ± 2.0 | 9.0 ± 1.7 | 25.1 ± 4.1 | 34.9 ± 11.3 | 10.3 ± 2.3 | | 53.6 ± 9.6 | 75.5 ± 17.7 |
| **Starch** | FpH18 | 10.5 ± 1.8 | 12.2 ± 5.4 | 31.3 ± 9.0 | 44.5 ± 8.7 | 12.2 ± 0.8 | | 92.6 ± 23.3* | 123.3 ± 30.0* |
|  | PsL2 | 9.7 ± 3.5 | 9.9 ± 3.8 | 50.2 ± 25.5 | 50.2 ± 9.8* | 11.7 ± 1.7 | | 69.7 ± 7.7* | 134.7 ± 27.4* |
|  | H18L2 | 8.9 ± 3.5 | 13.0 ± 3.4 | 34.6 ± 17.6 | 60.4 ± 21.5* | 13.0 ± 1.9 | | 78.7 ± 22.2* | 140.1 ± 7.8* |
|  | Col.0 | 143.3 ± 67.1 | 247.3 ± 112.0 | 278.5 ± 102.4 | 450.1 ± 76.8 | 355.9 ± 84.8 | | 397.5 ± 169.5 | 734.7 ± 101.5 |
| **3PGA** | FpH18 | 122.9 ± 28.7 | 235.6 ± 98.6 | 305.3 ± 139.9 | 423.2 ± 59.0 | 207.2 ± 22.3* | | 197.4 ± 86.4* | 538.4 ± 117.7* |
|  | PsL2 | 163.0 ± 56.3 | 277.0 ± 67.7 | 230.9 ± 101.7 | 319.9 ± 91.1* | 310.7 ± 145.8 | | 276.4 ± 129.1 | 487.2 ± 150.3* |
|  | H18L2 | 139.1 ± 54.2 | 230.8 ± 83.0 | 295.4 ± 108.8 | 183.5 ± 53.8* | 201.9 ± 145.9 | | 213.3 ± 113.3* | 377.6 ± 156.6* |
|  | Col.0 | 13820 ± 2150 | 16626 ± 1032 | 18739 ± 1493 | 18401 ± 949 | 23288 ± 1755 | | 26748 ± 5789 | 33599 ± 11470 |
| **Total AA´s** | FpH18 | 12068 ± 2697 | 16810 ± 1796 | 19263 ± 2607 | 20594 ± 1094* | 26999 ± 2307* | | 25601 ± 7366 | 21866 ± 5845* |
|  | PsL2 | 16699 ± 1284* | 18986 ± 1154* | 18925 ± 2951 | 22221 ± 2782* | 27276 ± 2699* | | 26516 ± 5945 | 28850 ± 3557* |
|  | H18L2 | 18365 ± 1547* | 19484 ± 2771* | 19250 ± 910 | 21499 ± 2108* | 24812 ± 4526 | | 28883 ± 5862 | 27645 ± 4148* |
|  | Col.0 | 0.081 ± 0.022 | 0.135 ± 0.034 | 0.130 ± 0.025 | 0.306 ± 0.058 | 2.991 ± 0.338 | | 2.996 ± 1.322 | 3.758 ± 0.709 |
| **Gly/Ser ratio** | FpH18 | 0.066 ± 0.009 | 0.109 ± 0.038 | 0.107 ± 0.026 | 0.107 ± 0.078 | 2.321 ± 0.578 | | 1.848 ± 0.708 | 3.937 ± 0.409 |
|  | PsL2 | 0.054 ± 0.004* | 0.100 ± 0.024 | 0.112 ± 0.012 | 0.111 ± 0.013* | 1.543 ± 0.868* | | 1.331 ± 0.356* | 1.987 ± 0.808* |
|  | H18L2 | 0.062 ± 0.011 | 0.104 ± 0.011 | 0.017 ± 0.037 | 0.176 ± 0.037* | 1.048 ± 0.675* | | 1.439 ± 1.155 | 1.609 ± 0.751* |
|  | Col.0 | 142954 ± 44081 | 195977 ± 11716 | 248222 ± 33039 | 267986 ± 32910 | 271002 ± 23939 | | 339074 ± 96873 | 369156 ± 70407 |
| **Total OA´s** | FpH18 | 207187 ± 40092* | 321888 ± 46694* | 374610 ± 91272* | 311688 ± 29506* | 342707 ± 34058* | | 335253 ±109373 | 330536 ± 103286 |
|  | PsL2 | 276765 ± 68558* | 298219 ± 29042* | 385166 ± 60797* | 352629 ± 68266* | 366074 ± 98594* | | 405009 ± 32778 | 405817 ± 51667 |
|  | H18L2 | 337708± 88591* | 322039 ± 65179* | 324204 ± 33435* | 428594 ± 56192* | 348636 ± 63573* | | 393947 ± 60421 | 384586 ± 66296 |
